# Intratumoral nanofluidic system enhanced tumor biodistribution of PD‐L1 antibody in triple‐negative breast cancer

**DOI:** 10.1002/btm2.10594

**Published:** 2023-09-15

**Authors:** Hsuan‐Chen Liu, Simone Capuani, Andrew A. Badachhape, Nicola Di Trani, Daniel Davila Gonzalez, Robin S. Vander Pol, Dixita I. Viswanath, Shani Saunders, Nathanael Hernandez, Ketan B. Ghaghada, Shu‐Hsia Chen, Elizabeth Nance, Ananth V. Annapragada, Corrine Ying Xuan Chua, Alessandro Grattoni

**Affiliations:** ^1^ Department of Nanomedicine Houston Methodist Research Institute Houston Texas USA; ^2^ University of Chinese Academy of Science (UCAS) Beijing China; ^3^ Department of Radiology Baylor College of Medicine Houston Texas USA; ^4^ Texas A&M University College of Medicine Bryan Texas USA; ^5^ Texas A&M University College of Medicine Houston Texas USA; ^6^ Department of Radiology Texas Children's Hospital Houston Texas USA; ^7^ Center for Immunotherapy Research Houston Methodist Research Institute Houston Texas USA; ^8^ Neal Cancer Center Houston Methodist Research Institute Houston Texas USA; ^9^ Department of Physiology and Biophysics Weill Cornell Medicine New York New York USA; ^10^ Department of Chemical Engineering University of Washington Seattle Washington USA; ^11^ Department of Bioengineering University of Washington Seattle Washington USA; ^12^ Department of Surgery Houston Methodist Hospital Houston Texas USA; ^13^ Department of Radiation Oncology Houston Methodist Hospital Houston Texas USA

**Keywords:** anti PD‐L1, biodistribution, CT, drug delivery, radiotherapy, TNBC, tumor microenvironment

## Abstract

Immune checkpoint inhibitors (ICI), pembrolizumab and atezolizumab, were recently approved for treatment‐refractory triple‐negative breast cancer (TNBC), where those with Programmed death‐ligand 1 (PD‐L1) positive early‐stage disease had improved responses. ICIs are administered systemically in the clinic, however, reaching effective therapeutic dosing is challenging due to severe off‐tumor toxicities. As such, intratumoral (IT) injection is increasingly investigated as an alternative delivery approach. However, repeated administration, which sometimes is invasive, is required due to rapid drug clearance from the tumor caused by increased interstitial fluid pressure. To minimize off‐target drug biodistribution, we developed the nanofluidic drug‐eluting seed (NDES) platform for sustained intratumoral release of therapeutic via molecular diffusion. Here we compared drug biodistribution between the NDES, intraperitoneal (IP) and intratumoral (IT) injection using fluorescently labeled PD‐L1 monoclonal antibody (αPD‐L1). We used two syngeneic TNBC murine models, EMT6 and 4T1, that differ in PD‐L1 expression, immunogenicity, and transport phenotype. We investigated on‐target (tumor) and off‐target distribution using different treatment approaches. As radiotherapy is increasingly used in combination with immunotherapy, we sought to investigate its effect on αPD‐L1 tumor accumulation and systemic distribution. The NDES‐treated cohort displayed sustained levels of αPD‐L1 in the tumor over the study period of 14 days with significantly lower off‐target organ distribution, compared to the IP or IT injection. However, we observed differences in the biodistribution of αPD‐L1 across tumor models and with radiation pretreatment. Thus, we sought to extensively characterize the tumor properties via histological analysis, diffusion evaluation and nanoparticles contrast‐enhanced CT. Overall, we demonstrate that ICI delivery via NDES is an effective method for sustained on‐target tumor delivery across tumor models and combination treatments.

**Translational Impact Statement**

Intratumoral administration of immunotherapeutics holds promise for enhancing treatment efficacy while minimizing off‐target effects of systemic dosing. We show that despite tumor heterogeneity, sustained broad tumor biodistribution can be attained via a nanofluidic implant, regardless of the simultaneous use of radiotherapy. Given the molecular transport phenotypes in murine solid tumors often mirror that of humans, including vascular permeability and interstitial fluid pressure, we anticipate translational relevance of our findings. Our results may pave the way for developing an effective clinical strategy for intratumoral immunotherapy.

## 1 INTRODUCTION

Traditionally, the standard of care for triple‐negative breast cancer (TNBC) consists of neoadjuvant chemotherapy followed by surgery, where only 30%–40% of patients achieve pathological complete response.^1^, ^2^ Despite aggressive therapy, TNBC has a high metastatic and recurrence rate and poor overall survival.^3^, ^4^, ^5^, ^6^ Immunological profiling of TNBC indicates the tumor microenvironment (TME) has higher level of lymphocyte infiltration, tumor mutational burden rate^7^, ^8^ and programmed cell death receptor ligand 1 (PD‐L1) expression than other breast cancer subtypes.^9^ Considering this, TNBC is likely to benefit from immunotherapy such as immune checkpoint inhibitors (ICI).^10^ Although controversial,^10^, ^11^, ^12^, ^13^ high PD‐L1 expression in TNBC is correlated with improved overall survival and favorable clinical outcomes.^14^, ^15^ In line with this, αPD‐L1 monoclonal antibody (mAB) in combination with nab‐paclitaxel is now included as the standard of care for treatment of PD‐L1‐positive advanced TNBC.^16^ On another note, radiotherapy is used to enhance ICI therapy by inducing immunogenic cell death.^17^, ^18^ In fact, radiotherapy is actively explored in combination with immunotherapy in clinical trials including for TNBC treatment,^17^ with favorable safety profile.^19^

Conventionally, the standard administration route for ICI is intravenous (IV) injection, which has well‐established serum pharmacokinetics and pharmacodynamics.^20^, ^21^ Due to systemic administration, mAbs circulate throughout the body, where non‐specific binding could occur and trigger off‐target effects.^22^ In addition, the mechanobiology^23^ and molecular compartmentalization^24^ of solid tumors can impair mAb penetration and intratumoral distribution.^25^, ^26^ As such, systemically administered drugs require scaled‐up dosage to achieve clinical effectiveness, which often results in systemic toxicities and a less than optimal therapeutic index.^22^, ^27^ Treatment‐related adverse events (TRAE) associated with systemic ICI therapy include neutropenia, nausea, anemia, interstitial pneumonitis, colitis with gastrointestinal perforation, and severe skin reactions.^28^, ^29^, ^30^, ^31^ These toxicities necessitate dose reduction or interruption, and thus affect ICI therapeutic efficacy.^32^, ^33^, ^34^, ^35^

On this note, intratumoral (IT) delivery is gaining momentum preclinically and clinically for immunotherapy administration.^36^, ^37^ IT administration delivers therapeutic mAbs directly into the tumor, achieving a high initial local drug concentration. However, due to bolus volume injection, drugs are rapidly cleared from the tumor requiring iterative treatment injections to achieve effective drug doses. This often entails invasive procedures due to factors such as tumor location and accessibility and carries risk of procedure‐associated infections. Further, drug leakage into systemic circulation generates toxicities conventional to systemic administration.^37^, ^38^ Thus, the optimal administration method for IT drug delivery remains undetermined.^39^, ^40^, ^41^

To resolve challenges related to repeated IT injections, as well as to enhance antitumor immune response and reduce the risk of systemic toxicity, we developed a long‐term sustained local drug release platform.^42^, ^43^, ^44^, ^45^, ^46^ Our IT drug delivery platform, termed the nanofluidic drug‐eluting seed (NDES), leverages a Si‐SiC‐based nanochannel membrane for continuous, controlled therapeutic release.^47^, ^48^ In a previous study, we demonstrated that sustained release of αCD40 and αPD‐L1 mAbs via the NDES significantly reduced 4T1 murine TNBC tumor burden, induced CD8+ T cells tumor infiltration, and most importantly, avoided TRAE compared to systemic delivery.^45^ We further showed that sustained IT release of αCD40 via the NDES improved local drug bioavailability with limited systemic dissemination.^44^ However, αPD‐L1 biodistribution in tumors with different PD‐L1 expression patterns in both tumor cells and tumor‐infiltrating immune cells is unknown. Moreover, there is a lack of knowledge on how these differences could impact IT αPD‐L1 administration, given the heterogeneity of tumor properties such as density and vascularity within the same cancer subtype.

In this study, we investigated the biodistribution of αPD‐L1 locally and systemically, comparing IP, direct IT bolus injection and sustained NDES diffusive delivery approach in two murine TNBC models. Fluorescently labeled αPD‐L1 were used to longitudinally analyze tumor and off‐target tissue dissemination differences between the three treatment delivery routes. Given that radiotherapy (RT) is increasingly investigated in the clinic with IT immunotherapy,^17^, ^18^, ^22^, ^49^, ^50^ we explored the impact of combining RT with αPD‐L1 on tumor retention and biodistribution. As drug delivery could be influenced by tumor cell density,^51^ we assessed the diffusion coefficient of αPD‐L1 in the two TNBC models using fluorescence recovery after photobleaching (FRAP).^52^ Further, in vivo nanoparticle contrast‐enhanced CT (nCECT) imaging was used, in combination with post‐mortem microscopic analysis, to examine differences in tumor vascular architecture and study the effects of RT in the two TNBC models.

## 2 MATERIALS AND METHODS

- Reagents: antagonist PD‐L1 (B7‐H1) monoclonal antibody (αPD‐L1) was purchased from BioXCell (BE0101; Lebanon, NH). Alex Fluor 700 NHS Ester (Succinimidyl Ester) dye was purchased from ThermoFisher Scientific (A20110; Waltham, MA). Purified Mouse anti‐Rat IgG2b heavy chain antibody, clone MARG2b‐3 (MCA1294) and purified mouse anti‐Rat Kappa/Lamda light chain antibody, clone MARK‐1/MARL‐15 (MCA1296P) were purchased from Bio‐Rad (Hercules, CA).
- Fluorescent antibody conjugation and lyophilization: αPD‐L1 was concentrated by centricon tubes (Millipore) with a molecular weight of 30 kDa. Filtrants were equilibrated with 0.1 M of NaHCO_3_ and incubated with AlexaFluor 700 (AF700) *N*‐Hydroxysuccinimide (NHS) ester on the rotor at 4°C overnight. The reaction mixture was concentrated and washed with PBS in centricon tubes with a molecular weight of 30 kDa. AF700 was selected due to its red/near‐infrared excitation and emission wavelength, which has the least impact from background signal of skin and fur. The concentrated PD‐L1‐AF700 was mixed with trehalose dehydrate with 37% w/w for drug stabilization during lyophilization. The mixture was gradually frozen with dry ice and lyophilized for 48 h with LABCONCO Freezone 4.5 (Hampton, NH).
- NDES fabrication: The fabrication of nanochannel membranes was previously published.^47^ The NDES device consists of a silicone‐based membrane with nanochannels and a cylindrical stainless‐steel drug reservoir (18G, 3.5 mm in length). The membrane and drug reservoir were affixed with implantable‐grade thermal epoxy (EPO‐TEK 354‐T) and cured overnight at 60°C. The reservoir was filled with lyophilized antibodies, then sealed with silicone adhesive (MED3‐4213, Nusil), and dried at 4°C overnight. To prevent drug leakage during the implantation, we applied UV epoxy over the silicone cap and cured under UV for about 15 s. To prepare for implantation, the NDES devices were weighed before and after priming with sterile PBS for drug release.
- TNBC tumor model: 4T1 (CRL02539) and EMT6 (CRL‐2755) murine mammary carcinoma cell lines were obtained from American Type Culture Collection and cultured according to manufactured culture protocol. Seven‐week‐old female BALB/c mice were purchased from Taconic Bioscience (Rensselaer, NY). Animals were housed at the Houston Methodist Research Institute Comparative Medicine under conditions outlined in the National Institutes of Health Guide for Care and Use of Laboratory Animals and Animal Care and Use Review Office (ACURO) and monitored daily. For in vivo imaging, mice were given alfalfa‐free diet to minimize background autofluorescence.
  For inoculation, low passage cells were resuspended at 3 × 10^4^ cells (4T1) or 5 × 10^5^ cells (EMT6) in 100 μl of 3:1 mixture of PBS and Matrigel matrix. The cell mixture was injected at the fourth left mammary fat pad. Tumor volume was monitored thrice weekly using the formula: 0.5 × Length × Width.^2^ Length was the longest axis of the tumor and width was perpendicular to the length.
  Treatment administration: Mice were randomized into designated groups (*n* = 6) when the size of the tumors reached 120 mm^3^. Single dose of 100 μg PD‐L1‐AF700 in PBS was given via intraperitoneal (IP; 100 μl) or IT (10 μl) injection. NDES was loaded with ~1 mg of lyophilized PD‐L1‐AF700 then primed for drug release. To prime the membrane for drug release, NDES was submerged in 1 ml of sterile phosphate‐buffered saline (PBS) under vacuum for 20 min. NDES was intratumorally implanted via a minimally invasive trocar approach.
- Radiation‐treated mice were anesthetized with inhaled isoflurane and intraperitoneal dexmedetomidine with 5 μg/g (body weight) before radiation. Radiation was administered using RS 2000 small animal irradiator (160 kV, 25 mA and 2 Gy/min mean beam rad source; Brentwood, TN). 4T1 tumors received 8 Gy while EMT6 tumors received 5 Gy for 3 consecutive days before immunotherapy treatment. A rigid exposed‐flank shield (Precision X‐ray Inc.) and a flexible lead layer were used to shield the mice from irradiation while exposing only the tumor. Anti‐sedative, atipamezole, was given to reverse dexmedetomidine anesthesia. To maintain the mice health post‐radiation, subcutaneous fluid and moist chow were given daily.
- In vivo and ex vivo fluorescent imaging: The tumor area on the mice was shaved before baseline image acquisition. Mice under isoflurane were fluorescently imaged for drug biodistribution using epi‐fluorescence on an in vivo bioluminescence/fluorescence imaging system (IVIS spectrum, Perkin Elmer) at designated time points. To detect AF700‐labeled mAbs, we used an excitation wavelength of 675 nm and an emission wavelength filter of 720 nm. The images were processed by Living Image Software (Perkin Elmer) by drawing a region of interest (ROI) around the tumor and measuring the fluorescent signal in radiance (p/s/cm/sr). Treatment naïve animals were used for subtracting baseline/background signal, and the scale bars were adjusted to the same setting for all experimental groups over 14 days. Experimental data were fitted with a double exponential using a nonlinear least squares fitting algorithm in MATLAB. The time constant τ was calculated by fitting the experimental data in (first timepoint excluded) with a single exponential in the form *ae* ^(*x*/τ)^. τ is the time at which the signal has decreased by 63.2% its initial value.
  For ex vivo imaging, mice were euthanized by CO_2_ asphyxiation and organs were harvested for imaging (*n* = 6 mice per group per timepoint). The same dimensions of ROI were applied around each organ to measure the radiance values. The fluorescence detection settings used were the same as in vivo fluorescent imaging.
- Quantification of in vivo PD‐L1‐AF700 biodistribution: Three tumors were randomly selected from each treatment group after ex vivo imaging. Each tumor was embedded in Tissue‐Tek OCT compound in plastic tissue embedding mold. The freezing procedure was described in a previous publication.^44^ Frozen tissues were sectioned and processed as described previously. Tumors were 3D reconstructed using a custom‐made MATLAB script.^53^, ^54^
- Anti‐Rat IgG enzyme‐linked immunosorbent assay (ELISA): Conjugated PD‐L1‐ AF700 Rat IgG was detected via an anti‐rat IgG sandwich ELISA. Serum was collected at designated time points. Thermo Scientific Pierce BCA Protein Assay Kit was used to determine tissue protein concentration and samples were adjusted to 1 μg/ml. 96‐well Nunc Maxisorp plates (Thermo Fisher) were coated with anti‐rat heavy chain antibody at 0.5 μg/ml (#MCA278, Bio‐Rad Laboratories, Hercules, CA, USA) overnight at 4°C. A standard curve was created with serial dilutions from PD‐L1 (B7‐H1) monoclonal antibody from BioXCell (BE0101; Lebanon, NH). The secondary antibody was an anti‐rat kappa/lambda light chain antibody conjugated to horseradish peroxidase (#MCA1296P, Bio‐Rad Laboratories). 1‐Step UltraTMB (Thermo Fisher) was used as substrate and the colorimetric reaction was stopped with ELISA Stop Solution (Invitrogen). Plates were analyzed for absorbance at 450 nm in a UV–Vis plate reader (BioTek Synergy H4).
- Flow cytometry: PD‐L1 expression level on TNBC cell line surface was assessed via flow cytometry. 4T1 and EMT6 cells were single‐cell suspended in 96 well U‐bottom plates. 1 × 10^6^ cells were incubated with PE anti‐mouse CD274 (B7‐H1, BioLegend, 124308) at 4°C for 20 min, washed with PBS twice, then resuspended in PBS containing 2% of fetal bovine serum. Cells were acquired by LSRII (BD Bioscience), and the flow data were analyzed using FlowJo v10 software. Acquisition excluded debris, doublets and nonviable cells.
- Histopathology analysis of tumor cell density, vasculature, and collagen: Tumors were formalin‐fixed and paraffin‐embedded (FFPE). Masson's trichrome staining of 5–6 tumor sections per tumor model was performed for collagen area analysis. Eight regions of interest (ROI) from each section were analyzed via color deconvolution script on MATLAB 2021b. For vasculature assessment, 5–6 tumor sections per tumor model were stained with anti‐αSMA (ab5694, Abcam), followed by goat anti‐Rabbit Alexa Flour 555 (A27039, ThermoFisher Scientific), and DAPI counterstain for nuclei. CD45 and PD‐L1 staining was performed on *n* = 5 slides per condition using anti‐CD45 (14‐0451‐82, eBiosciences) and anti‐CD274 (14‐5982‐82, eBiosciences) monoclonal antibodies followed by mouse anti‐rat IgG2b (50‐4815‐82, eBiosciences) and goat anti‐rat IgG2a (PA1‐84761, Invitrogen) secondary antibodies and DAPI counterstain for nuclei. Eight images per section were taken at ×40 magnification with a confocal microscope (Olympus FV3000). Cell counting and blood vessel area fraction evaluation were performed with Olympus CellSens. DAPI staining was used for cellular density analysis. CD45, DAPI, and PD‐L1 colocalization was quantified with QuPath 0.4.3.
  Diffusion measurement by fluorescence recovery after photobleaching (FRAP) analysis: 4T1 and EMT6 tumors were harvested when tumor size reached 150 mm^3^ from BALB/c mice. We cross‐sectioned the tumors and incubated the tumor section with Dulbecco's Modified Eagle Medium containing 10% of fetal bovine serum and penicillin and streptomycin. αPD‐L1 was labeled with fluorescein‐5‐isothiocyanate (FITC, F1906, Thermo Fisher Scientific) and separated with Zeba spin desalting columns (89891, Thermo Fisher Scientific). αPD‐L1‐FITC was measured and incubated with tumor sections with 1 mg/ml of final concentration in culture media for 24 h in 8‐well chamber slide (154453, Thermo Fisher Scientific). The simulation approach for photobleaching was performed on Olympus FluoView FV3000 confocal microscope, the setting followed manufactural protocol and reference.^55^, ^56^, ^57^ Briefly, an intense laser beam is used to bleach a defined region containing fluorescently labeled antibody. The bleached area and nearby area (background) were identified as ROI and monitored prior to and after photobleaching over a designated period of time. We randomly selected three 4T1 and EMT6, 6–8 ROIs from center and edge of each tumor. After bleaching, the neighboring (non‐bleached) molecules diffused into the area. FRAP analysis was performed using CellSens imaging software (Olympus). The recovery data was fitted to an exponential equation with background photobleaching correction to obtain the characteristic diffusion time τ. The diffusion coefficient is determined by the following equation.

(1) $$D=\frac{r^{2}}{4\tau }$$

where *r* is the radius of the circular beam.^58^

- Nanoparticle contrast‐enhanced computed tomography (nCECT) imaging: Computed tomography (CT) imaging was performed on a small animal micro‐CT system (Siemens Inveon). Animals were sedated with 3% isoflurane, setup on the CT animal bed, and then maintained at 2%–2.5% isoflurane delivered via nose cone. The scan parameters for the CT image acquisition were: 50 kVp, 0.5 mA, 850 ms X‐ray exposure, 540 projections, 44 μm isotropic spatial resolution, scan time ~ 20 min. A long circulating liposomal‐iodine (Lip‐I) contrast agent was used for nanoparticle contrast‐enhanced CT (nCECT) imaging.^59^, ^60^, ^61^ Animals underwent a pre‐contrast scan followed by intravenous injection of Lip‐I contrast agent (1.65 mg I/kg body weight) administered through either tail vein or retro‐orbital route. Delayed nCECT imaging was performed 7 days post‐Lip‐I injection to examine vascular leakiness of the tumor. A second dose of Lip‐I was then immediately administered to acquire an acute nCECT scan for tumor vascular imaging. All datasets were Hounsfield Unit (HU) calibrated for image analysis. All image analysis was performed in Osirix (version 5.8.5, 64‐bit, Pixmeo, Bernex, Switzerland). CT‐derived tumor FBV (tFBV) was calculated as the ratio of signal enhancement in tumor to signal enhancement in the blood (inferior vena cava, *IVC*) between delayed nCECT and acute nCECT scans as per the following equation^59^, ^62^, ^63^, ^64^:

(2) $$\text{tFBV}=\frac{HU_{T,\text{acute}}-HU_{T,\text{delay}}\;}{HU_{\mathrm{IVC},\text{acute}}-HU_{\mathrm{IVC},\text{delay}}}$$

where ${\mathrm{HU}}_{T}$ indicates mean CT signal (expressed in HU) for segmented tumor volume and ${\mathrm{HU}}_{\mathrm{IVC}}$ indicates mean CT signal for region of interest (ROI) drawn in the IVC. Tumor vascular leak (tVL) was evaluated by computing a normalized tumor leak score. The tumor leak score was computed by measuring CT signal enhancement in the tumor relative to signal enhancement in the liver (primary organ for clearance of Lip‐I). A normalization approach was implemented to account for inter‐animal variability in contrast injection. Pre‐contrast and delayed nCECT scans were used to compute CT signal enhancement in tumor and liver as per the following equation^63^, ^65^:

(3) $$\mathrm{tVL}=\frac{HU_{T,\text{delay}}-HU_{T,\mathrm{pre}}\;}{HU_{L,\text{delay}}-HU_{L,\mathrm{pre}}}$$

- Where ${\mathrm{HU}}_{T}$ indicates mean CT signal in segmented tumor volume and ${\mathrm{HU}}_{L}$ indicates mean CT signal in liver. A total of 12 mice underwent nCECT imaging (*n* = 6 EMT6 mice and *n* = 6 4T1 mice). In each cohort of EMT6 and 4T1 mice, three mice underwent radiation therapy, and three mice were used as control (no radiation therapy). All mice were implanted with bilateral flank tumors. One EMT6 mouse died prior to acute‐contrast scan. Mann–Whitney test was used for statistical analysis of quantitative nCECT metrics. A *p* value <0.05 was considered statistically significant.
- Statistical analysis: Statistical analysis was performed in GraphPad Prism 9.1.2. For multiple group comparisons, an analysis of variance (ANOVA) was performed. Unpaired 1‐way, 2‐way ANOVA and multiple comparisons were performed using Tukey corrections. *p* < 0.05 was considered significant.

## 3 RESULTS AND DISCUSSION

### 3.1 NDES

**FIGURE 1:**
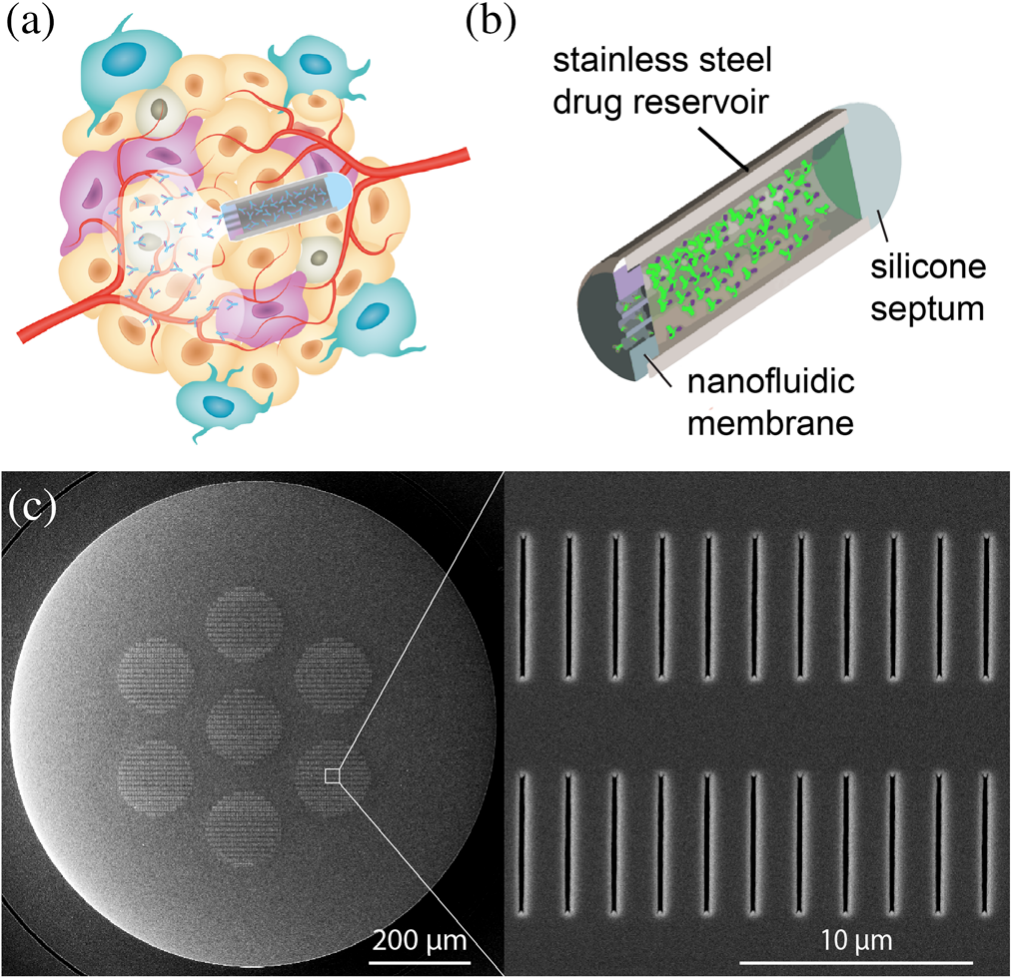
Rendering of NDES and SEM pictures of nanofluidic membrane. (a) Rendering of intratumoral NDES releasing mAbs. (b) Longitudinal cross‐section rendering of NDES showing different components. (c) SEM image of NDES membrane (left) and higher magnitude image of the slit‐nanochannel array (right).

Abnormal physiological conditions in the TME present a physical barrier that limits drug penetration through extravasation, convection, and diffusion.^66^ To overcome this, we developed the NDES for drug release directly into the tumor in a sustained and constant diffusive manner (Figure 1a). The NDES platform consists of a stainless steel cylindrical drug reservoir, in which a silicon nanofluidic membrane is affixed on one end (Figure 1b). The nanofluidic membrane structure includes seven microchannels, each directly connected to a dense array of 1400 through slit‐nanochannels (Figure 1c). Drug release across the membrane occurs through concentration‐driven diffusive transport, whereby the release rate is dependent on electrostatic and steric hindrance effects when confined within the nanochannels.^67^, ^68^ As such, NDES achieves sustained and constant delivery without relying on mechanics or pumping mechanisms. The biocompatibility of the device and materials were previously demonstrated in small and large animal models.^45^, ^69^, ^70^ The NDES is designed for a one‐time minimally invasive IT implantation procedure using a clinical trocar approach similar to brachytherapy seed insertion.

### 3.2 Distinct αPD‐L1 mAb TME biodistribution profiles associated with administration routes and tumor models

In this study, we used two orthotopic syngeneic murine TNBC models, 4T1 and EMT6, to assess the biodistribution of PD‐L1‐AF700 when administered systemically or locally. Both cell lines expressed PD‐L1, with 4T1 cells having a lower cell surface expression level than EMT6 cells (Figure S1). Moreover, 4T1 is characterized as a poorly immunogenic model, whereas EMT6 is highly immunogenic.^71^, ^72^

**FIGURE 2:**
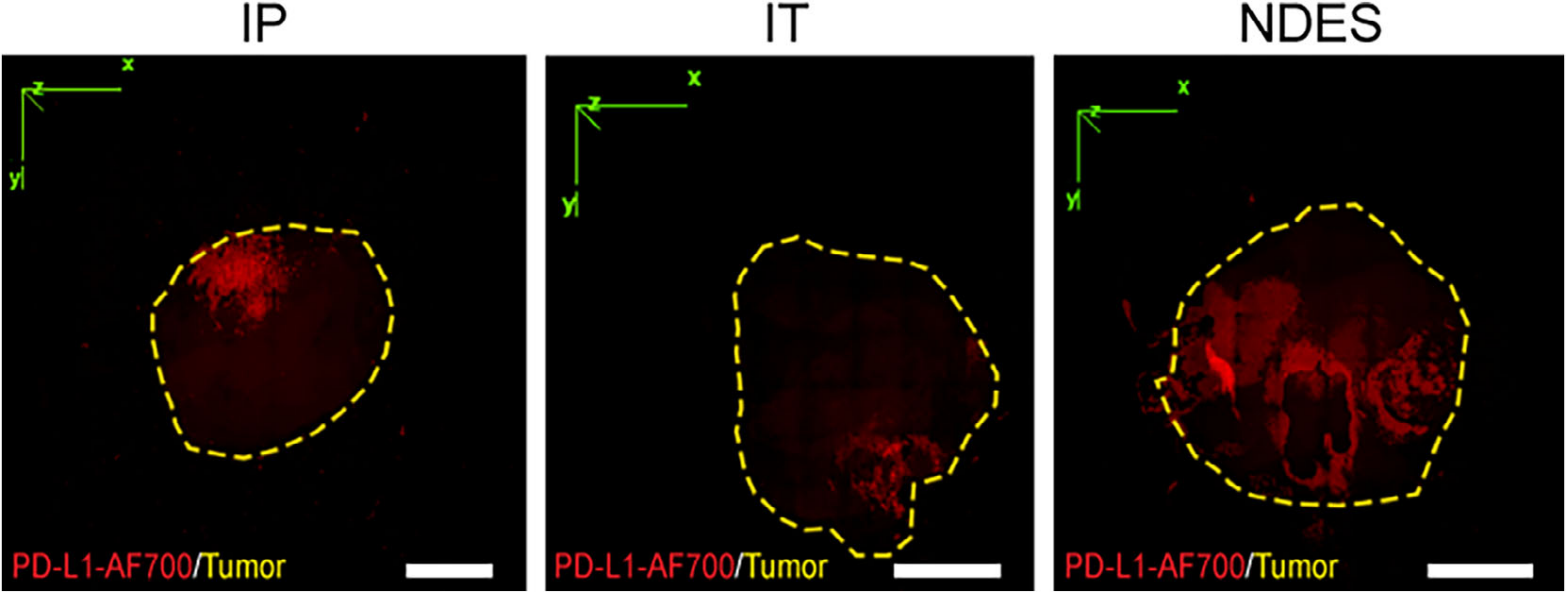
Representative 3D rendering images of normalized antibody distribution within tumor via IP, IT, and NDES delivery methods. PD‐L1‐AF700 antibody (red) showed biodistribution within 4T1 tumors (yellow dotted line outlined the tumor volume) 7 days post administration. Scale bar 2 mm.

To assess biodistribution, we used Alexa Fluor 700 (AF700) to fluorescently label αPD‐L1 (αPD‐L1‐AF700). Fluorescence labeling allows for drug detection via immunofluorescence imaging of histological tumor sections as well as in vivo real‐time visualization and quantification via radiant efficiency measurements using in vivo imaging system (IVIS) software. As a preliminary comparative evaluation of routes of administration, we first used the 4T1 mouse model. Tumor‐bearing mice were each randomized into three treatment arms: IP injection, direct IT injection and NDES. A single dose of 100 μg αPD‐L1‐AF700 was given via IP or bolus IT injection. αPD‐L1‐AF700 loaded NDES devices with release rates of ~7.6 ± 1.5 μg per day (cumulative release over 14 days of 105.8 ± 20.5 μg)^45^, ^46^ were implanted intratumorally in a one‐time minimally invasive procedure. Seven days post‐administration, we evaluated αPD‐L1‐AF700 distribution and retention within the tumor using immunofluorescence imaging. 3D‐reconstructed imaging of the entire tumor showed that NDES cohort had a more homogenous biodistribution within the tumor compared to IP or IT delivery (Figure 2).

**FIGURE 3:**
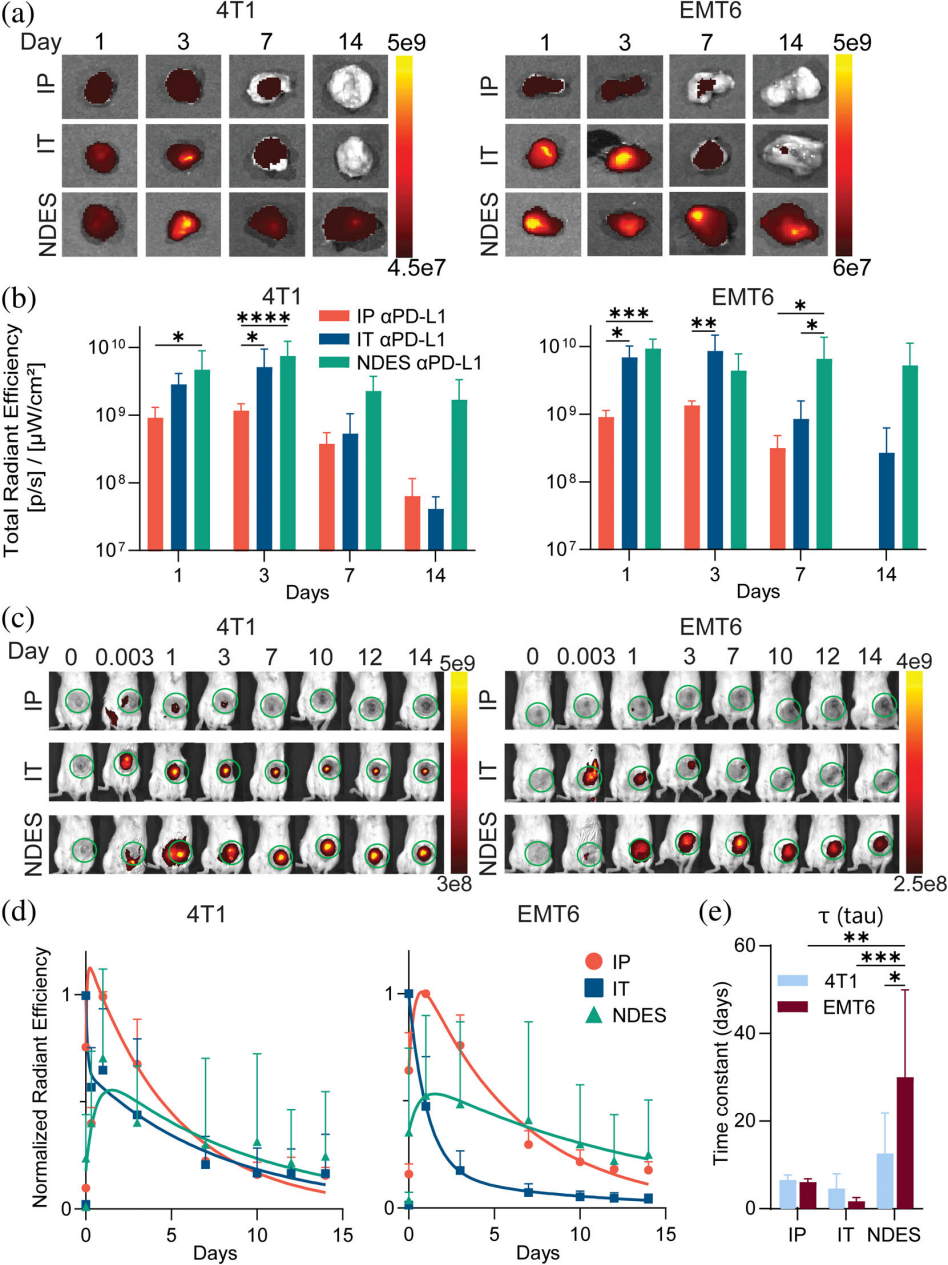
In vivo and ex vivo analyses of αPD‐L1‐AF700 tumor biodistribution via IP, IT and NDES delivery methods. (a) Ex vivo IVIS imaging of αPD‐L1‐AF700 on days 1, 3, 7, and 14. (b) Bar graph depicts the radiance signal measured. Two‐way ANOVA was performed for statistical analysis. (c) In vivo monitoring of αPD‐L1‐AF700 over 14 days via IVIS live animal imaging. Green circle denotes the tumor and radiance signal measurement area. (d) Exponential fitting analysis of αPD‐L1‐AF700 normalized radiant efficiency measurement within tumors. (e) Time constant (τ) representing the decay rate of αPD‐L1‐AF700 from the tumor. One‐way ANOVA was performed for statistical analysis. *p < 0.05; **p < 0.005; ***p < 0.0005; ****p < 0.0001 (*n* = 6 for each analysis).

Next, we tracked the drug biodistribution and retention in the tumor over time using IVIS in both 4T1 and EMT6 mice. Mice were administered with αPD‐L1‐AF700 using the three delivery routes as described above. To avoid confounding artifacts related to animal fur, skin and surrounding organs, tumor was harvested for evaluation of αPD‐L1‐AF700 via ex vivo radiant efficiency measurement over the course of 14 days (Figure 3a, b). Sustained presence of αPD‐L1‐AF700 was evident in both models of the NDES groups throughout the study duration, while the IP group showed low αPD‐L1‐AF700 signal, indicating modest tumor penetration. Although tumors in the IT group had high levels of αPD‐L1‐AF700 upon bolus injection, signal depletion occurred after day 3, suggesting rapid clearance.

An additional group of animals was used to non‐invasively and longitudinally track the local pharmacokinetic (PK) of αPD‐L1‐AF700 at frequent time points. Mice were imaged on the ventral view to monitor αPD‐L1‐AF700 signal via IVIS over 2 weeks (Figure 3c). While IVIS signal in vivo can vary from animal to animal based on depth of tumor, animal position and confounding effects of skin and fur, it allows longitudinal assessment of drug concentration for each individual animal. Thus, rather than comparing absolute signal values (Figure S2), here we focused on comparing the time evolution of radiant efficiency profiles among the three groups. For this, the signals measured longitudinally for each animal were normalized to its own maximum value. Average normalized datasets were then obtained for each group (Figure 3d). The larger dataset allowed us to reliably obtain exponential fitting of the normalized radiant efficiency for each group (*R* ^2^ > 0.88 for all groups) and the related time constant (τ), which quantifies how rapidly αPD‐L1‐AF700 concentration decayed in the TME (Figure 3e).

For the IP group, in both 4T1 and EMT6 cohorts, the peak of signal occurs around day 1, followed by a rapid decay highlighted by the low τ value. A similar trend is observed in the IT group in both models; in this case, the maximum signal was detected earlier, consistent with the direct local IT injection. τ analysis indicated that upon IT injection, αPD‐L1‐AF700 decayed faster in EMT6 compared to 4T1. Consistent with the in vivo evaluation, both models in the NDES group had extended presence of drug in the tumor throughout the study in comparison with the other administration routes. IVIS signal variation is more marked for the NDES group, which can be explained by the variability in the NDES positioning within the tumor, thus potentially blocking the signal. In fact, the tumors imaged ex‐vivo, after NDES removal, have similar standard deviations to the ones in the IT group. One order of magnitude higher τ values were obtained with NDES as compared to IT and IP bolus injections. Further, τ analysis for NDES showed longer retention in EMT6 compared to 4T1, indicating slowed clearance from the TME.

As later discussed, we posit that the differences in drug penetration between the models and administration routes could be attributed to the heterogeneous characteristics of the TME in solid tumors, including extracellular matrix density, leaky vasculature, and high interstitial fluid pressure (IFP). In the IP cohorts, extravasation from circulatory vasculature and penetration into the tumor could be impeded by the TME barrier. Tumor density could impact diffusivity within the tumor, and in turn, drug localization and retention. In the case of the IT group, a bolus volume injection could rapidly leak out of denser tumors due to high IFP, resulting in lower retention. On the contrary, steady molecular diffusion from the NDES, which is associated with the delivery of a negligible volume of drugs, results in higher local retention, which is further augmented in TME with higher PD‐L1 expression, such as in the case of EMT6.

### 3.3 Radiation influenced the αPD‐L1‐AF700 biodistribution in 4T1 and EMT6 TNBC murine model

Radiotherapy can induce immunogenic cell death as well as alter the TME, such as blood vessels permeability, immune cells infiltration and ECM composition,^73^, ^74^, ^75^ further enhancing anti‐tumor immune responses. Moreover, radiation can remodel tumor vasculature and enhance vascular permeability of small molecules.^76^ The tumor vascular permeability is also significantly variable depending on tumor type.^77^ Further, some tumors have elevated IFP owing to the high vascular permeability.^78^

**FIGURE 4:**
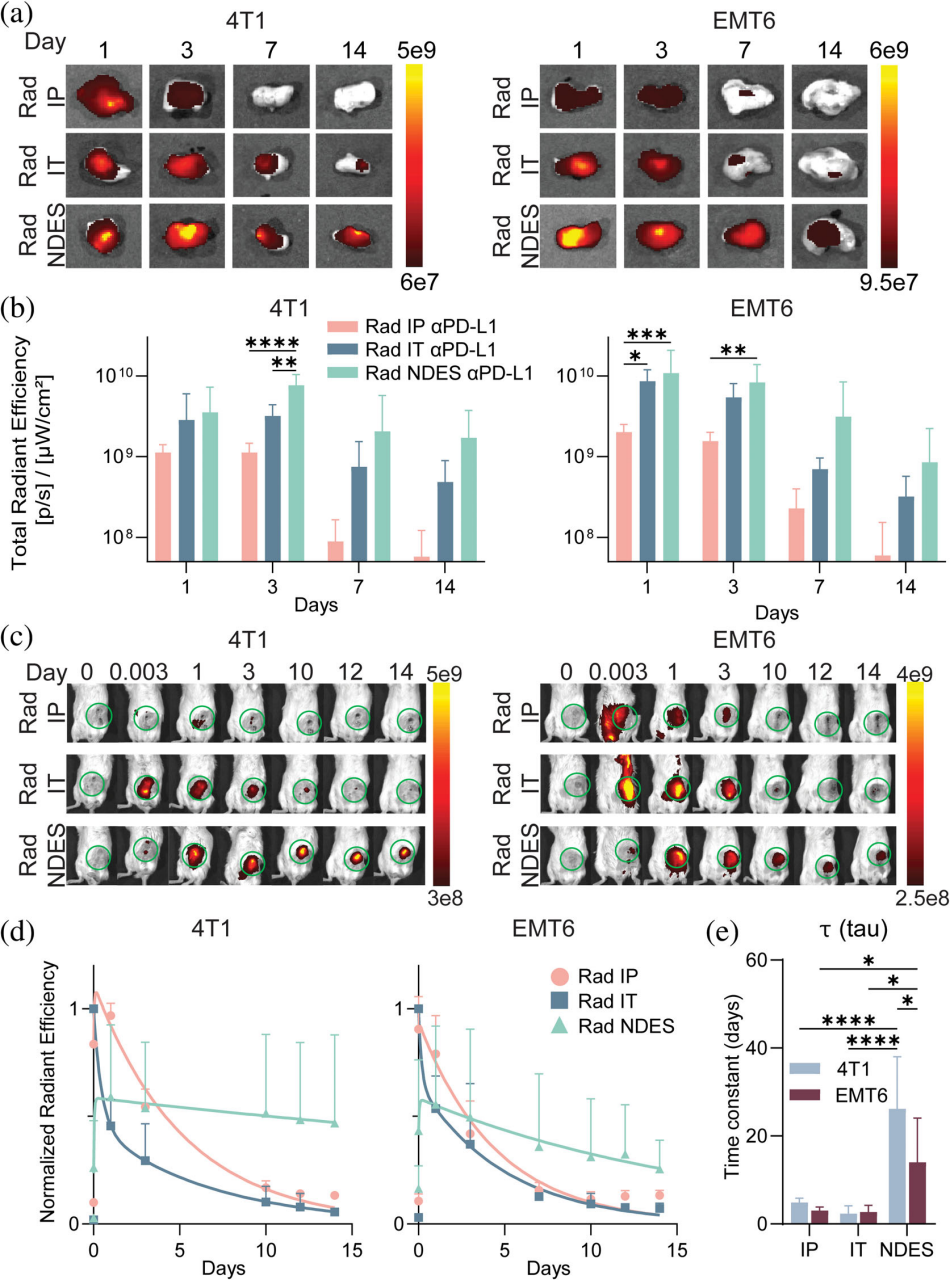
In vivo and ex vivo imaging analyses of the radiated tumors over 14 days after αPD‐L1‐AF700. (a) Ex vivo IVIS imaging of αPD‐L1‐AF700 on day 1, 3, 7 and 14. (b) Bar graph depicts the radiance signal measured. Two‐way ANOVA was performed for statistical analysis. (c) In vivo monitoring of αPD‐L1‐AF700 over 14 days. (d) Exponential fitting analysis of αPD‐L1‐AF700 normalized radiant efficiency within tumors. (e) Time constant (τ) representing the decay rate of αPD‐L1‐AF700 from the tumor. One‐way and two‐way ANOVA was performed for statistical analysis. *p < 0.05; **p < 0.005; ***p < 0.0005; ****p < 0.0001 (*n* = 6 for each analysis).

To determine the influence of radiation on αPD‐L1 biodistribution, 4T1 and EMT6 tumors were irradiated prior to αPD‐L1‐AF700 administration via three different routes. αPD‐L1‐AF700 biodistribution in radiated tumors was monitored via ex vivo and in vivo IVIS imaging. Similar to non‐radiated tumors, IP route exhibited low drug penetration and limited retention in both 4T1 and EMT6 (Figure 4a, b). Likewise, radiation pre‐treatment followed by IT injection did not impact tumor distribution and retention when comparing 4T1 and EMT6. In contrast, NDES group showed sustained presence of αPD‐L1‐AF700 in the radiated 4T1 tumors, as opposed to EMT6, which was reflected in the normalized radiant efficiency trends and τ analysis (Figure 4c–e). Interestingly, the radiance peak is shifted to earlier timepoint for the IP groups in both radiated tumor models.

**FIGURE 5:**
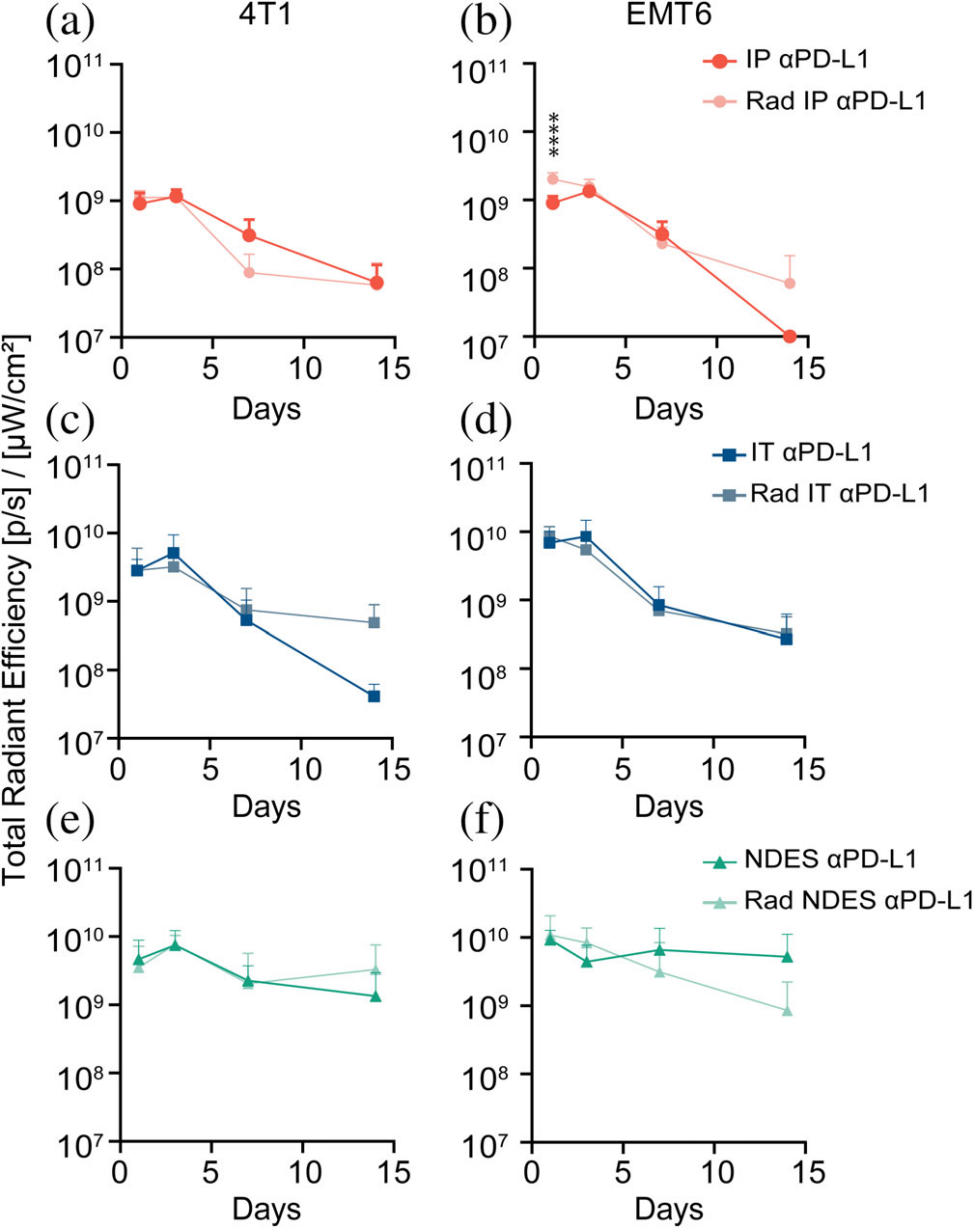
Ex vivo fluorescence imaging analysis of the tumors over 14 days after αPD‐L1‐AF700 administration. Line graphs depict radiance signal (*n* = 6 per group) from IP delivery of (a) 4T1 and (b) EMT6 tumors, IT delivery of (c) 4T1 and (d) EMT6 tumors, and NDES delivery of (e) 4T1 and (f) EMT6 tumors. Two‐way ANOVA was performed for statistical analysis. Statistical markers refer to individual timepoints, ****p < 0.0001 (*n* = 6 for each analysis).

In addition, we compared the radiant efficiency signal between non‐radiated and radiated tumors across the different delivery approaches (Figure 5). In IP‐treated 4T1 tumors, radiation did not affect intratumoral αPD‐L1‐AF700 distribution (Figure 5a). On the contrary, EMT6 cohort showed significant uptake of αPD‐L1‐AF700 within the tumor in radiated IP group compared to non‐radiated on day 1 (Figure 5b). This finding suggests potential radiation‐induced vascular damage resulting in increased vascular permeability and extravasation, thus supporting higher tumor uptake for IP αPD‐L1‐AF700 delivery. Notably, retention appears to be enhanced in the radiated 4T1 tumors for both the IT and NDES delivery, while the opposite is observed in the EMT6 model with NDES administration (Figure 5c–f). We speculate that radiation treatment lead to enhanced immunogenicity in the 4T1 tumor resulting in an increase in immune cell infiltration and overall density, which in turn could reduce αPD‐L1 clearance. Further, radiation could increase surface expression of PD‐L1 on tumor cells,^79^, ^80^, ^81^ resulting in augmented αPD‐L1 binding.

### 3.4 4T1 and EMT6 TNBC models have distinct tumor properties

**FIGURE 6:**
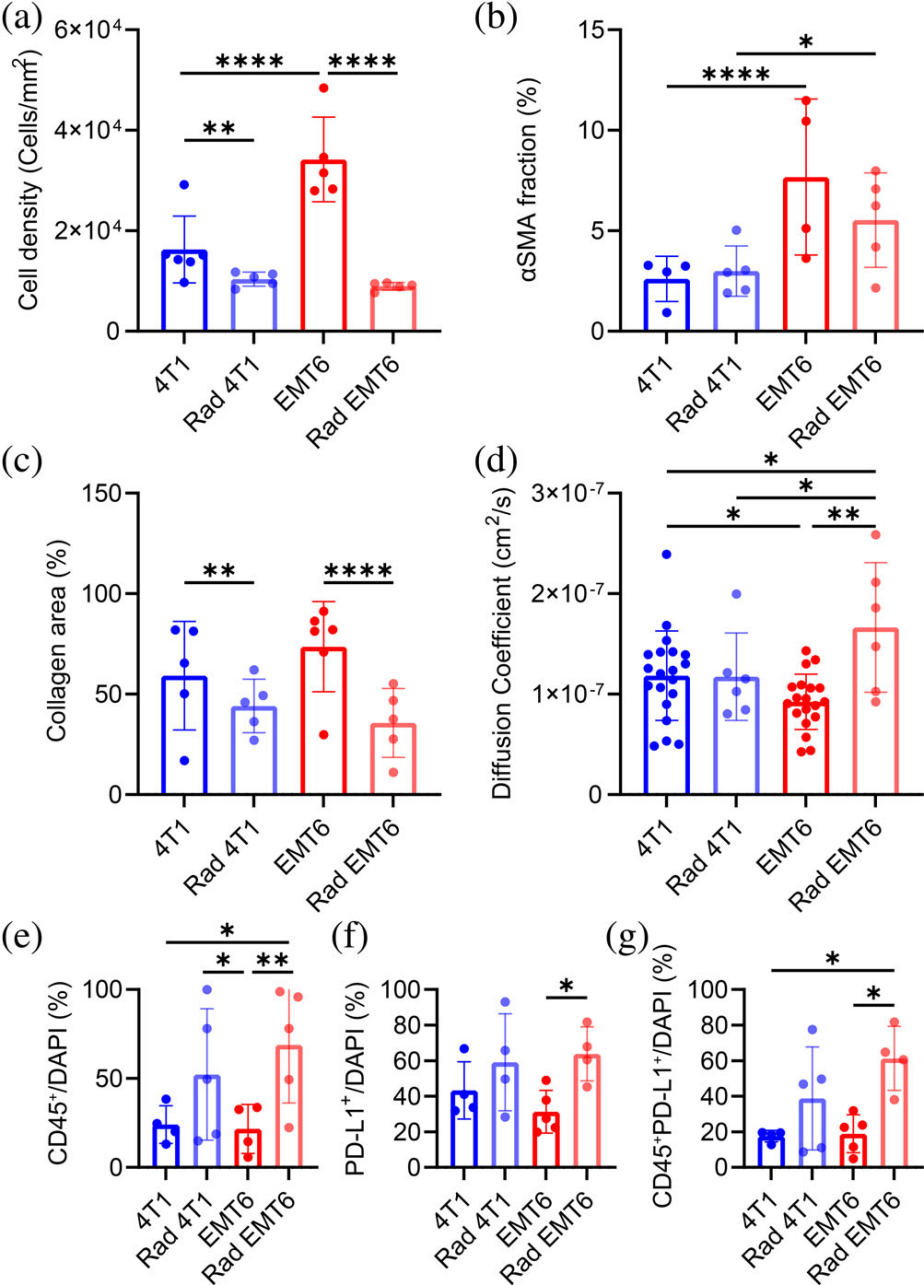
Tumor properties assessment of murine TNBC models, 4T1 and EMT6. Quantification of (a) cellular density (*n* = 5), (b) αSMA fraction (*n* = 4), and (c) collagen fraction (*n* = 6) in 4T1 and EMT6 tumors non‐radiated and radiated. (d) Diffusion coefficients of 4T1 and EMT6 as determined via FRAP analysis (*n* = 20 non‐radiated, *n* = 6 radiated). Ten ROIs were analyzed from three tumors of each TNBC model. Quantification of (e) CD45^+^, (f) PD‐L1^+^, and (g) CD45^+^PD‐L1^+^ cells in tumor slides (*n* = 5). Two‐tailed unpaired *t*‐test was performed for statistical analysis. *p < 0.05; **p < 0.005; n.s., no significance.

To better understand the findings above, we investigated the correlation between mAb biodistribution and tumor properties, by evaluating the tumor density and vasculature of 4T1 and EMT6. EMT6 showed significantly higher cell density and vasculature compared to 4T1 (Figures 6a, b and S3) and no difference in collagen content (Figures 6c and S3). After the radiation treatment there was a drop in cell and collagen density, likely caused by radiation mediated cell‐death. Next, we assessed the diffusion coefficients of FITC‐labeled αPD‐L1 mAb in 4T1 and EMT6 radiated and non‐radiated tumors using fluorescence recovery rate after local photobleaching (FRAP; Figure S4). FRAP evaluation provides critical information on the diffusion of molecules within solid tumors. Specifically, this experiment was performed to evaluate the interstitial transport of the antibody and impact of the physical TME. FRAP analysis revealed significantly lower diffusion coefficient in EMT6 compared to 4T1 model (Figure 6d). The slow molecule diffusion within EMT6 tumors could be attributable to high cellular density, which in turn affects drug penetration and accumulation.^82^ In line with this, we noted slower αPD‐L1‐AF700 clearance from the EMT6 tumors (τ), as compared to 4T1 (Figure 3). EMT6 tumors subjected to radiation pre‐treatment showed a significantly higher diffusion coefficient compared to non‐radiated EMT6 tumors coinciding with a nearly 2‐fold decrease in τ value (Figure S5). The increase in retention observed in radiated 4T1 tumors is not mirrored by a decrease in the diffusion coefficient, discarding physical modification of the TME as a justification for such behavior. Immunohistochemistry analysis revealed an overall increase in immune cells infiltration in radiated tumors (Figure 6e). In this context, the higher number of PD‐L1 expressing cells could explain the enhanced retention observed in the 4T1 radiated tumors (Figure 6f, g).

### 3.5 nCECT revealed radiation therapy altering the tumor properties

To further elucidate the impact of radiation therapy on tumor properties between the two syngeneic TNBC models, nanoparticle contrast‐enhanced CT (nCECT) imaging was performed in 4T1 and EMT6 tumor‐bearing mice with and without radiation therapy. nCECT is a non‐invasive and quantitative technique that enables in vivo whole‐tumor interrogation of tumor vascular density and permeability.^60^, ^62^, ^83^ nCECT imaging is also used to study the effects of radiation therapy on tumor vasculature.^63^, ^65^

**FIGURE 7:**
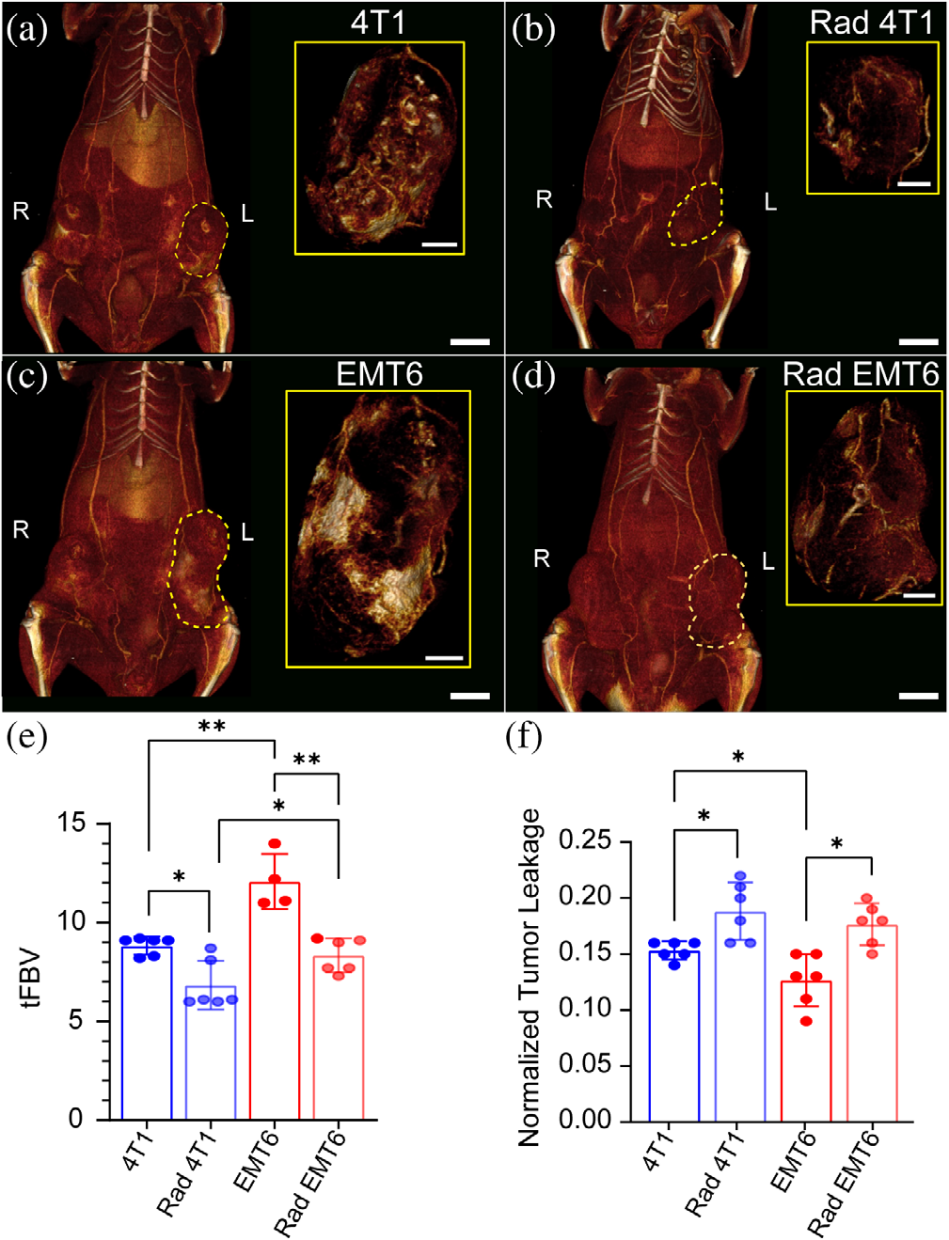
Nanoparticle contrast‐enhanced CT (nCECT) imaging of tumor vasculature in 4T1 and EMT6 models. Whole body high‐resolution 3D volume rendered images of (a) non‐radiated and (b) radiated 4T1 tumor‐bearing mice, and (c) non‐radiated and (d) radiated EMT6 tumor‐bearing mice. Scale bar 5 mm, inset scale bar 2 mm. (e) nCECT‐derived tumor fractional blood volume (tFBV), and (f) normalized tumor leakage. Statistical analysis was performed using a Mann–Whitney *U* test. *p < 0.05; **p < 0.005 (*n* = 6 for each analysis except for the fFBV assessment of the EMT6 where there was *n* = 4).

To evaluate the impact of radiation therapy on αPD‐L1 biodistribution, 4T1 and EMT6 tumor‐bearing mice were treated with three consecutive radiation doses of 8 and 5 Gy, respectively (Figure 7a–d). Radiation dosages were chosen based on literature according to tumor model and our preliminary unpublished data.^84^ Whole body high‐resolution 3D volume rendered images showed that non‐radiated 4T1 tumors have a denser vascular network than radiated cohorts (Figure 7a, b, inset). Similar trends were observed between non‐radiated and radiated EMT6 tumors (Figure 7c, d, inset). Consistent with histological analysis (Figure 6b), nCECT‐derived tumor fractional blood volume (tFBV), an indicator of tumor vascular density, was significantly lower in non‐radiated 4T1 (8.8% ± 0.5%) compared to EMT6 (12.1% ± 1.4%) tumors (Figure 7e). nCECT‐derived normalized tumor leakage, an indicator of tumor vascular permeability, was significantly lower in non‐radiated EMT6 (0.13 ± 0.02) compared to 4T1 (0.15 ± 0.03) (Figure 7f). nCECT imaging further revealed that radiation therapy markedly reduced tFBV in both 4T1 (6.8% ± 1.2%) and EMT6 (8.3% ± 0.9%) tumors (Figure 7e). On the contrary, radiation increased tumor leakage scores of both 4T1 (0.19 ± 0.03) and EMT6 (0.18 ± 0.02) (Figure 7f). These data suggested that radiation therapy results in tumor vascular damage, leading to increased vascular permeability and higher extravasation of nanoparticle contrast agent.

In summary, consistent with the histology imaging analysis, nCECT imaging revealed distinct differences in tumor vascular architecture between the two TNBC models and the impact of radiation pre‐treatment.

### 3.6 NDES showed minimal αPD‐L1 dissemination to other organs

Systemic administration of therapeutics usually accumulates in non‐targeted organs, leading to dose‐limiting toxicities.^85^, ^86^, ^87^ Further, capping at the maximum tolerated dosage also limits antitumor treatment efficacy.^88^ In a previous study of αCD40 mAb biodistribution, we observed prevalent liver accumulation with systemic IP administration.^44^ We posit that liver accumulation contributed to toxicities and adverse effects in systemic‐treated mice.^43^, ^45^, ^46^

Here, we collected liver, lung, kidney, spleen, and inguinal lymph node (LN) from radiated and non‐radiated 4T1 and EMT6 mice at designated time points to assess αPD‐L1‐AF700 presence via ex vivo IVIS imaging (Figures S6–S11). Radiance values of each organ were presented in bar graphs for visualization of αPDL1‐AF700 biodistribution from three different delivery routes over 14 days (Figures S12 and S13).

**FIGURE 8:**
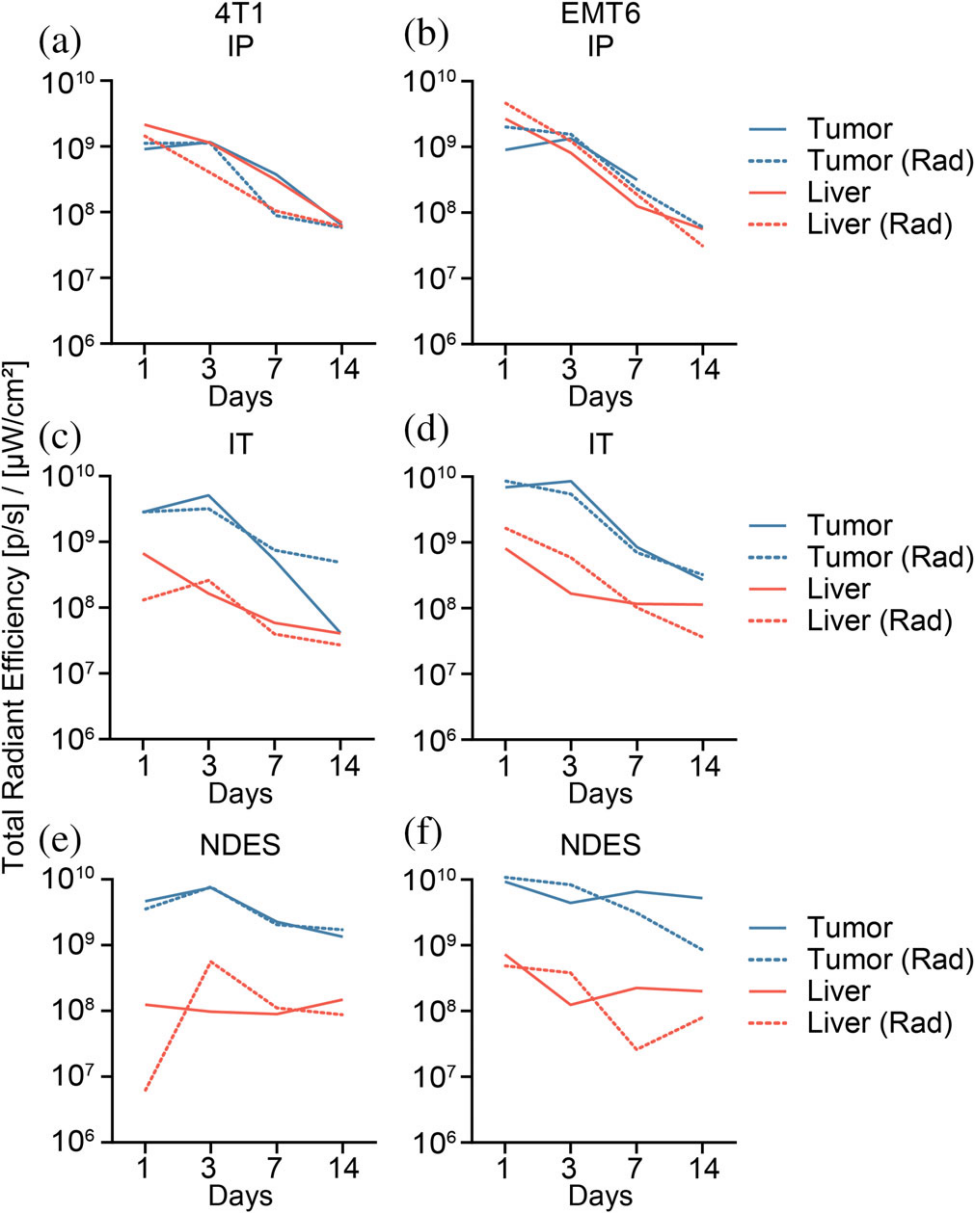
Ex vivo fluorescence analysis of tumor (blue) and liver (red) across 14 days. αPD‐L1‐AF700 signal was compared across different delivery routes in 4T1 and EMT6 tumors: (a, b) IP, (c, d) IT, and (e, f) NDES, either radiated (dotted line) or non‐radiated (solid line). Line graphs depict the total radiance signal measured.

As the liver is associated with ICI‐induced toxicities,^89^ we selectively assessed αPD‐L1‐AF700 liver biodistribution in comparison to tumor. In both murine models, IP group showed comparable levels of αPD‐L1‐AF700 in the liver and tumor for both non‐radiated (solid line) and radiated (dotted line) cohorts (Figures 8a,b and S12 and S13). This data highlights the off‐target concerns of systemic delivery, further supported by high serum levels of αPD‐L1 mAb one day after administration (Figures S14). In contrast, both IT and NDES cohorts showed presence of αPD‐L1‐AF700 in the liver, albeit at ~1.0–1.5 orders of magnitude lower than tumor (Figure 8c–f). The serum levels of αPD‐L1 mAb one day after administration in IT cohorts (except for radiated 4T1) suggest rapid tumor leakage (Figure S14), which is consistent with clinical reports of systemic dissemination from bolus volume IT injection.^41^, ^90^, ^91^, ^92^, ^93^ Contrarily, sustained release achieved through passive diffusion with NDES circumvented this effect. Therefore, αPD‐L1 clearance plausibly depended on passive interstitial transport and vascular intravasation,^94^ resulting in minimal serum and organs levels. Overall, the biodistribution study of αPD‐L1‐AF700 supports the premise that systemic delivery can have substantial off‐target effects, which cannot be sufficiently addressed by bolus IT injection.

## 4 CONCLUSION

In conclusion, our study shows that sustained IT diffusive dosing via the NDES can significantly improve ICI tumor retention and minimize off‐target biodistribution. Most importantly, we observed that tumor model and combined treatments such as radiation can have a significant impact on ICI biodistribution. Higher antibody clearance was observed in non‐radiated 4T1 tumors compared to EMT6, and was corroborated by the differences in the physical characteristics of the tumors. After radiation, both tumor models had a decrease in vascularity and an increase in leakiness of the remaining vessels. In line with these observations and a significant increment of the diffusion coefficient, retention in the radiated EMT6 tumor substantially dropped. In contrast, radiation had an opposite effect on 4T1 tumors, improving the retention of αPD‐L1 within the tumor compared to its non‐radiated counterpart. However, no notable change in the diffusion coefficient is observed. We speculate that the reduced clearance in radiated 4T1 tumors could be caused by changes in the immunological TME that led to an increase in PDL1‐expressing cells. In light of this, adaptable drug‐agnostic platforms like the NDES can provide a highly versatile approach for the treatment of TNBC with minimal off‐target dissemination.

## AUTHOR CONTRIBUTIONS

**Hsuan‐Chen Liu:** Conceptualization (equal); data curation (lead); formal analysis (lead); investigation (lead); methodology (equal); validation (equal); visualization (equal); writing – original draft (lead). **Simone Capuani:** Conceptualization (equal); data curation (equal); formal analysis (equal); investigation (equal); methodology (equal); validation (equal); visualization (equal); writing – review and editing (lead). **Andrew A. Badachhape:** Data curation (supporting); formal analysis (supporting); methodology (supporting); validation (supporting); visualization (supporting); writing – review and editing (supporting). **Nicola Di Trani:** Formal analysis (supporting); visualization (supporting). **Daniel Davila Gonzalez:** Methodology (supporting). **Robin S. Vander Pol:** Data curation (supporting); methodology (supporting). **Dixita I. Viswanath:** Methodology (supporting). **Shani Saunders:** Methodology (supporting). **Nathanael Hernandez:** Methodology (supporting). **Ketan B. Ghaghada:** Methodology (supporting); validation (supporting); writing – review and editing (supporting). **Shu‐Hsia Chen:** Funding acquisition (supporting); validation (supporting). **Elizabeth Nance:** Formal analysis (supporting); funding acquisition (supporting); methodology (supporting); visualization (supporting). **Ananth V. Annapragada:** Resources (supporting); validation (supporting). **Corrine Ying Xuan Chua:** Conceptualization (equal); funding acquisition (equal); project administration (equal); supervision (equal); writing – review and editing (equal). **Alessandro Grattoni:** Conceptualization (equal); funding acquisition (equal); project administration (equal); supervision (equal); writing – review and editing (equal).

## CONFLICT OF INTEREST STATEMENT

Alessandro Grattoni is an inventor of intellectual property licensed by Semper Therapeutics. The other authors declare no conflict of interest.

### PEER REVIEW

The peer review history for this article is available at https://www.webofscience.com/api/gateway/wos/peer-review/10.1002/btm2.10594.

## Supporting information


**FIGURE S1.** PD‐L1 expression profile of TNBC murine models.
**FIGURE S2.** αPD‐L1‐AF700 absolute radiant efficiency measurement within (a) non‐radiated and (b) radiated tumors.
**FIGURE S3.** Histology images of tumor tissue acquired at ×40 magnification (Scale bar 50 μm).
**FIGURE S4.** FRAP sequences and analysis. (a) FRAP sequences of non‐radiated tumor tissue saturated with αPD‐L1 labeled with FITC (Image size 1200 μm). (b) FRAP recovery curves of non‐radiated tumors. (c) FRAP sequences of radiated tumor tissue saturated with αPD‐L1 labeled with FITC (Image size 1200 μm). (d) FRAP recovery curves of radiated tumors.
**FIGURE S5.** Time constant (τ) representing the decay rate of αPD‐L1‐AF700 from the tumor. (a) Time constant in 4T1 tumors. (b) Time constant in EMT6 tumors. Two‐way ANOVA was performed for statistical analysis *p < 0.05.
**FIGURE S6.** Ex vivo fluorescence imaging analysis of the organs from 4T1 (left) and EMT6 (right) mice over 14 days after αPD‐L1‐AF700. (a) Representative livers from each time points and bar graph depicts radiance signal measured. (b) Representative lungs from each time points and bar graph depicts radiance signal measured 2way ANOVA was performed for statistical analysis. *p < 0.05; **p < 0.005; ***p < 0.001; ****p < 0.0001.
**FIGURE S7.** Ex vivo fluorescence imaging analysis of the organs from 4T1 (left) and EMT6 (right) mice over 14 days after αPD‐L1‐AF700. (a) Representative kidneys from each time points and bar graph depicts radiance signal measured. (b) Representative spleens from each time points and bar graph depicts radiance signal measured 2way ANOVA was performed for statistical analysis. *p < 0.05; **p < 0.005; ***p < 0.001; ****p < 0.0001.
**FIGURE S8.** Ex vivo fluorescence imaging analysis of the organs from 4T1 (left) and EMT6 (right) mice over 14 days after αPD‐L1‐AF700. Representative LNs from each time points and bar graph depicts radiance signal measured. 2way ANOVA was performed for statistical analysis.*p < 0.05; **p < 0.005; ***p < 0.001; ****p < 0.0001.
**FIGURE S9.** Ex vivo fluorescence imaging analysis of the organs from 4T1 (left) and EMT6 (right) mice over 14 days after αPD‐L1‐AF700. (a) Representative livers from each time points and bar graph depicts radiance signal measured. (b) Representative lungs from each time points and bar graph depicts radiance signal measured 2way ANOVA was performed for statistical analysis. *p < 0.05; **p < 0.005; ***p < 0.001; ****p < 0.0001.
**FIGURE S10.** Ex vivo fluorescence imaging analysis of the organs from 4T1 (left) and EMT6 (right) mice over 14 days after αPD‐L1‐AF700. (a) representative kidneys from each time points and bar graph depicts radiance signal measured. (b) representative spleens from each time points and bar graph depicts radiance signal measured 2way ANOVA was performed for statistical analysis. *p < 0.05; **p < 0.005; ***p < 0.001; ****p < 0.0001.
**FIGURE S11.** Ex vivo fluorescence imaging analysis of the organs from 4T1 (left) and EMT6 (right) mice over 14 days after αPD‐L1‐AF700. (a) representative LNs from each time points and bar graph depicts radiance signal measured. 2way ANOVA was performed for statistical analysis. *p < 0.05; **p < 0.005; ***p < 0.001; ****p < 0.0001.
**FIGURE S12.** Biodistribution of αPDL1‐AF700 across different organs over 14 days. Ex vivo fluorescence analysis of the tumor, liver, lung, kidneys, spleen, and inguinal LN were analyzed by measuring the radiance signal at the end of sacrificing point of each delivery method. Bar graphs depict radiance signal measured. Each data point represents mean ± STD (*n* = 5–6 per organ).
**FIGURE S13.** Biodistribution of αPDL1‐AF700 across different organs over 14 days. Ex vivo fluorescence analysis of the tumor, liver, lung, kidneys, spleen, and inguinal LN from radiated mice were analyzed by measuring the radiance signal at the end of sacrificing point of each delivery method. Bar graphs depict radiance signal measured. Each data point represents mean ± STD (*n* = 5–6 per organ).
**FIGURE S14.** ELISA analysis of serum αPD‐L1 levels of non‐radiated and radiated 4T1 and EMT6 mice 1 day after administered via IP, IT, or NDES. Each data point represents mean ± STD (*n* = 6 per group).

## Data Availability

The data supporting this study's findings are available from the corresponding authors upon reasonable request.
