# Supplementary material for: Intratumoral nanofluidic system enhanced tumor biodistribution of PD‐L1 antibody in triple‐negative breast cancer
Source: Bioeng Transl Med. 2023 Sep 15;8(6):e10594. doi: 10.1002/btm2.10594 (PMC10658527; doi:10.1002/btm2.10594)
Supplement: Supplementary file 1 — FIGURE S1. PD‐L1 expression profile of TNBC murine models. FIGURE S2. αPD‐L1‐AF700 absolute radiant efficiency measurement within (a) non‐radiated and (b) radiated tumors. FIGURE S3. Histology images of tumor tissue acquired at ×40 magnification (Scale bar 50 μm). FIGURE S4. FRAP sequences and analysis. (a) FRAP sequences of non‐radiated tumor tissue saturated with αPD‐L1 labeled with FITC (Image size 1200 μm). (b) FRAP recovery curves of non‐radiated tumors. (c) FRAP sequences of radiated tumor tissue saturated with αPD‐L1 labeled with FITC (Image size 1200 μm). (d) FRAP recovery curves of radiated tumors. FIGURE S5. Time constant (τ) representing the decay rate of αPD‐L1‐AF700 from the tumor. (a) Time constant in 4T1 tumors. (b) Time constant in EMT6 tumors. Two‐way ANOVA was performed for statistical analysis *p < 0.05. FIGURE S6. Ex vivo fluorescence imaging analysis of the organs from 4T1 (left) and EMT6 (right) mice over 14 days after αPD‐L1‐AF700. (a) Representative livers from each time points and bar graph depicts radiance signal measured. (b) Representative lungs from each time points and bar graph depicts radiance signal measured 2way ANOVA was performed for statistical analysis. *p < 0.05; **p < 0.005; ***p < 0.001; ****p < 0.0001. FIGURE S7. Ex vivo fluorescence imaging analysis of the organs from 4T1 (left) and EMT6 (right) mice over 14 days after αPD‐L1‐AF700. (a) Representative kidneys from each time points and bar graph depicts radiance signal measured. (b) Representative spleens from each time points and bar graph depicts radiance signal measured 2way ANOVA was performed for statistical analysis. *p < 0.05; **p < 0.005; ***p < 0.001; ****p < 0.0001. FIGURE S8. Ex vivo fluorescence imaging analysis of the organs from 4T1 (left) and EMT6 (right) mice over 14 days after αPD‐L1‐AF700. Representative LNs from each time points and bar graph depicts radiance signal measured. 2way ANOVA was performed for statistical analysis.*p < 0.05; **p < 0.005; ***p < [file BTM2-8-e10594-s001.docx]

**Supplementary information**

**Intratumoral nanofluidic system enhanced tumor biodistribution of PD-L1 antibody in triple-negative breast cancer**

Hsuan-Chen Liu, Simone Capuani, Andrew A. Badachhape, Nicola Di Trani, Daniel Davila Gonzalez, Robin Vander Pol, Dixita I Viswanath, Shani Saunders, Nathanael Hernandez, Ketan B. Ghaghada, Shu-Hsia Chen, Elizabeth Nance, Ananth V. Annapragada, Corrine Ying Xuan Chua*, Alessandro Grattoni*

*Corresponding author


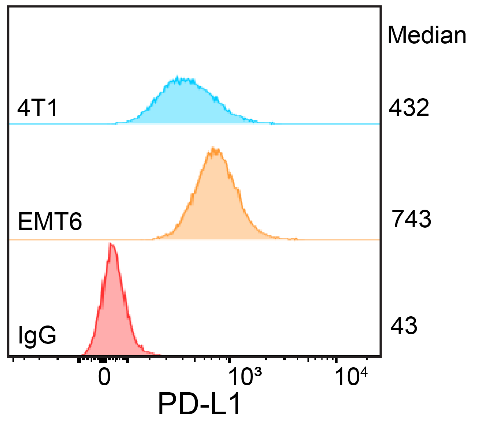


**sFig 1.** PD-L1 expression profile of TNBC murine models.


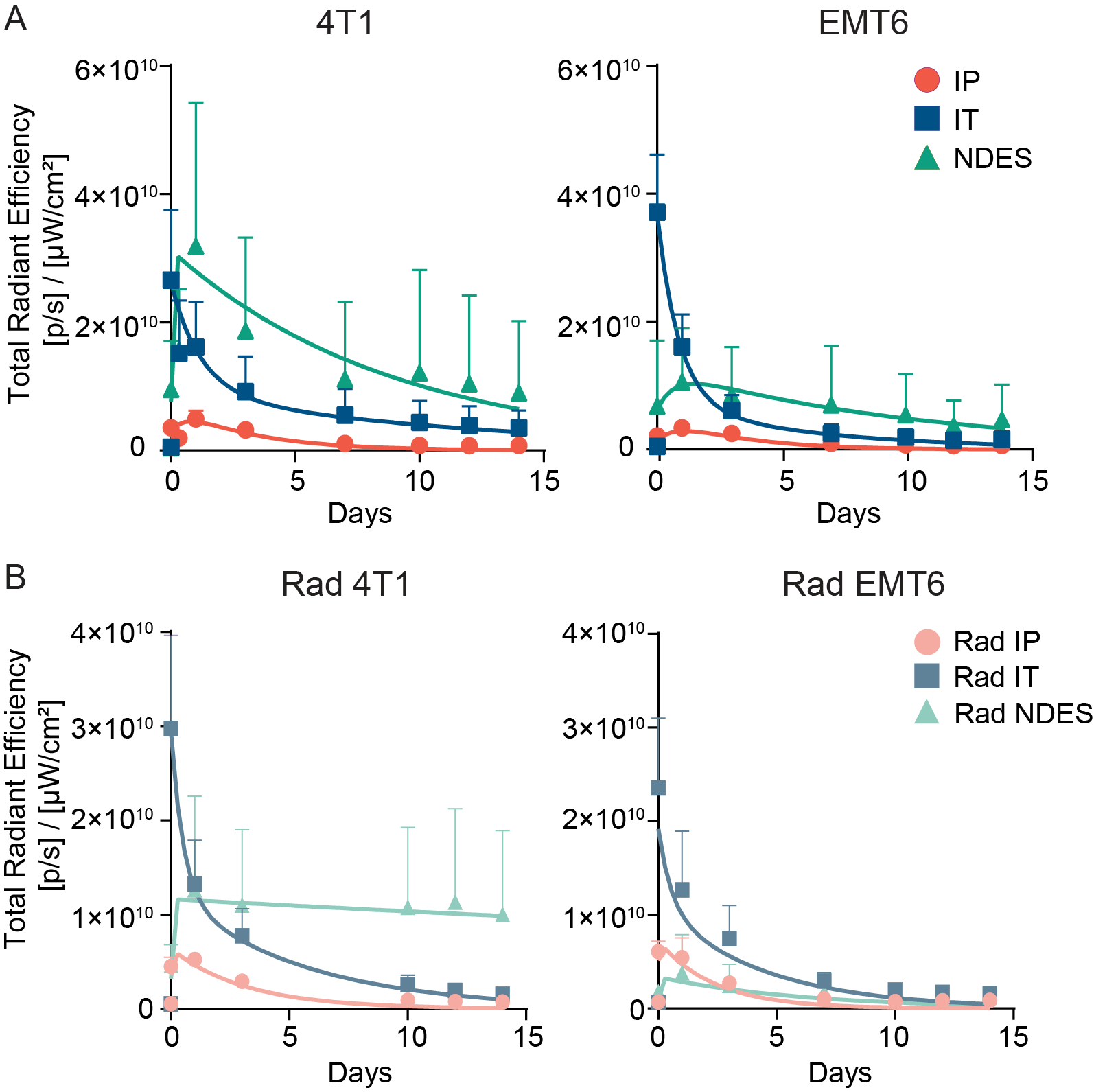


**sFig 2.** αPD-L1-AF700 absolute radiant efficiency measurement within A) non-radiated and B) radiated tumors


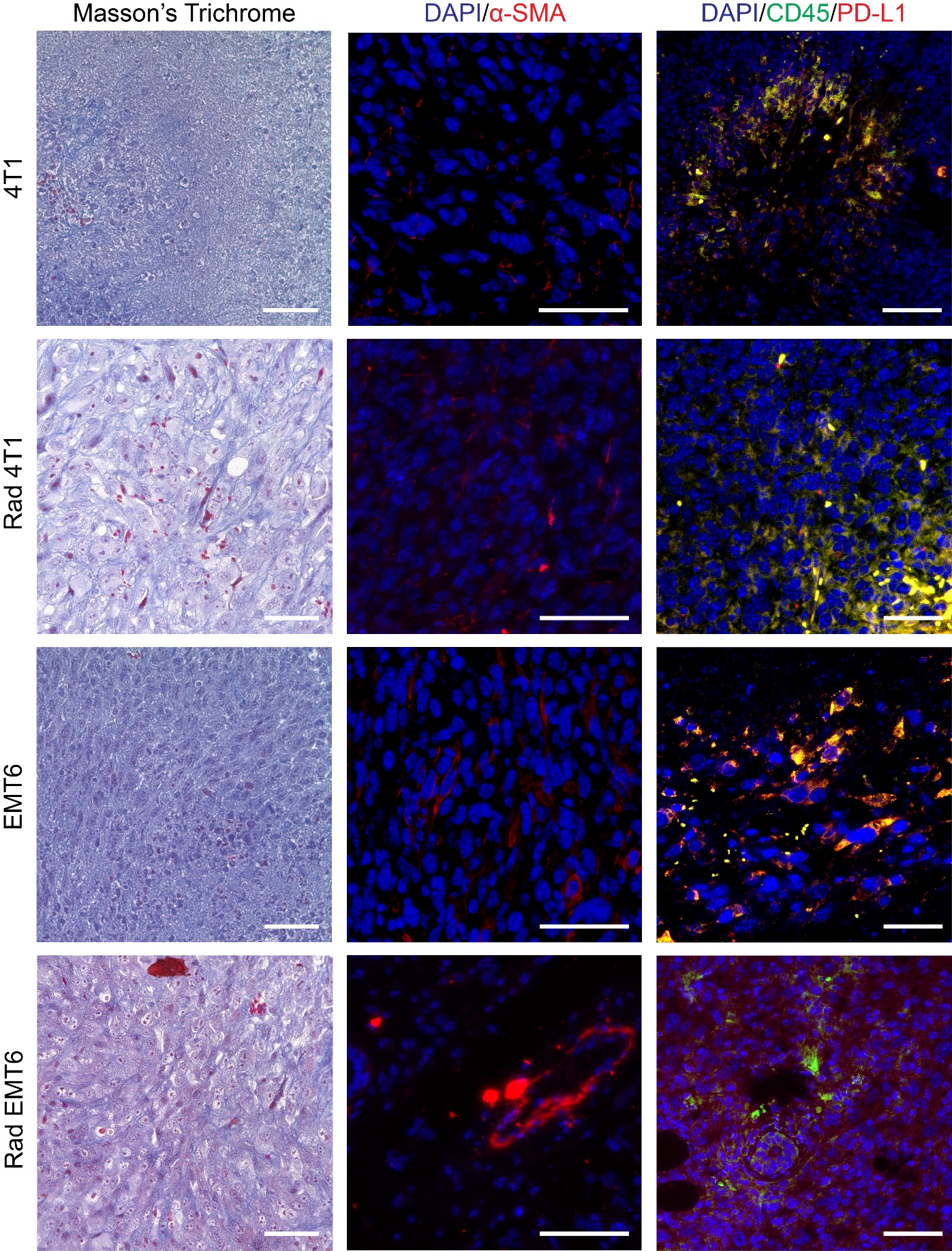


**sFig 3.** Histology images of tumor tissue acquired at 40x magnification (Scale bar 50 um).


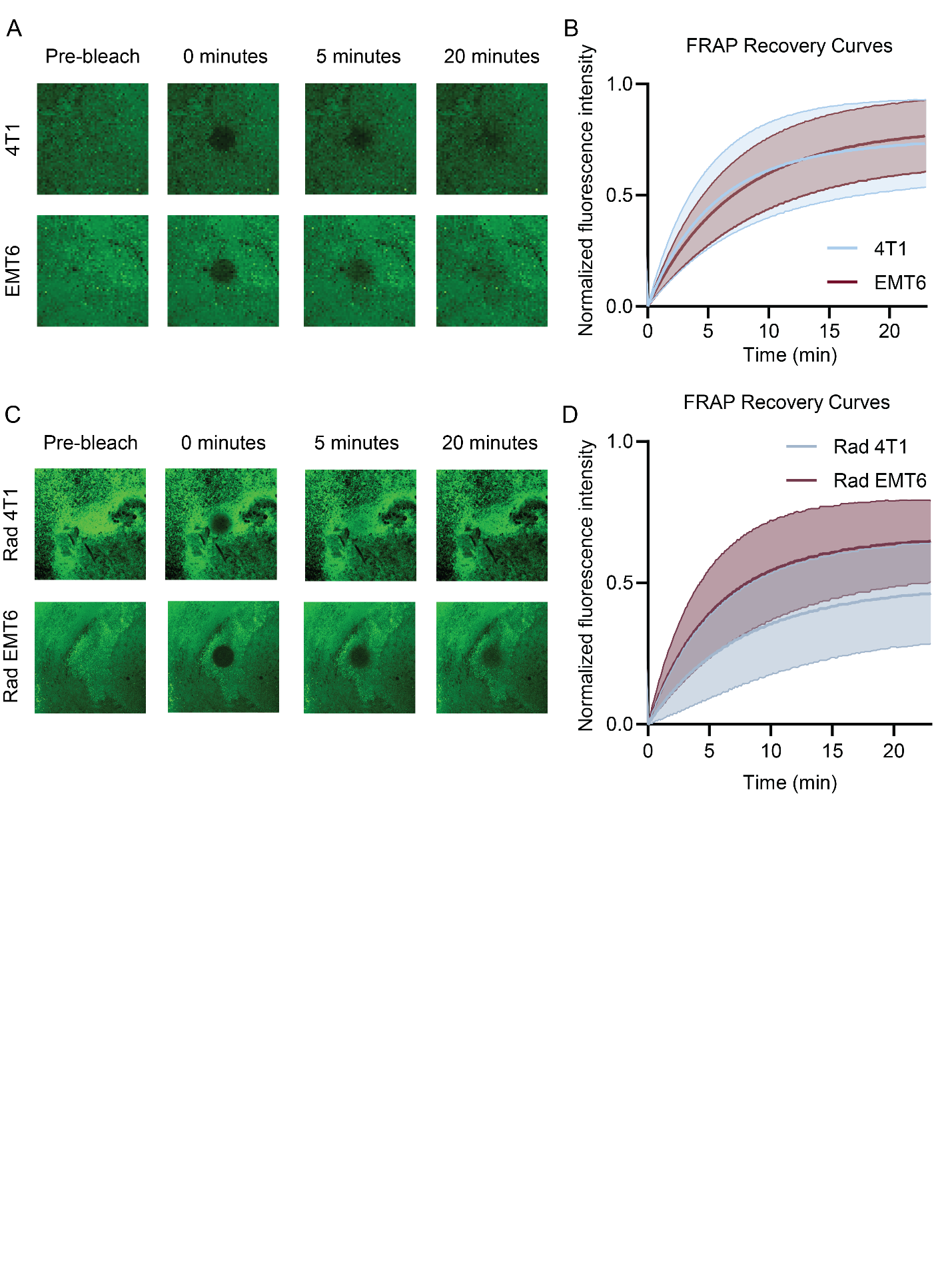


**sFig 4.** FRAP sequences and analysis. A) FRAP sequences of non-radiated tumor tissue saturated with αPD-L1 labeled with FITC (Image size 1200 μm). B) FRAP recovery curves of non-radiated tumors. C) FRAP sequences of radiated tumor tissue saturated with αPD-L1 labeled with FITC (Image size 1200 μm). D) FRAP recovery curves of radiated tumors.

**
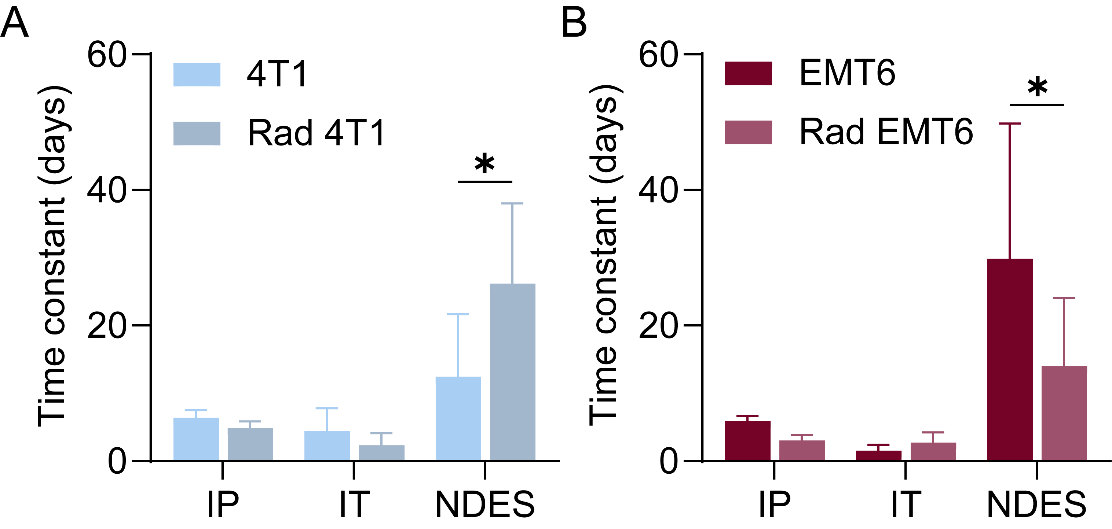
**

**sFig 5.** Time constant (τ) representing the decay rate of αPD-L1-AF700 from the tumor. A) Time constant in 4T1 tumors. B) Time constant in EMT6 tumors. Two-way ANOVA was performed for statistical analysis. p<0.05, *.


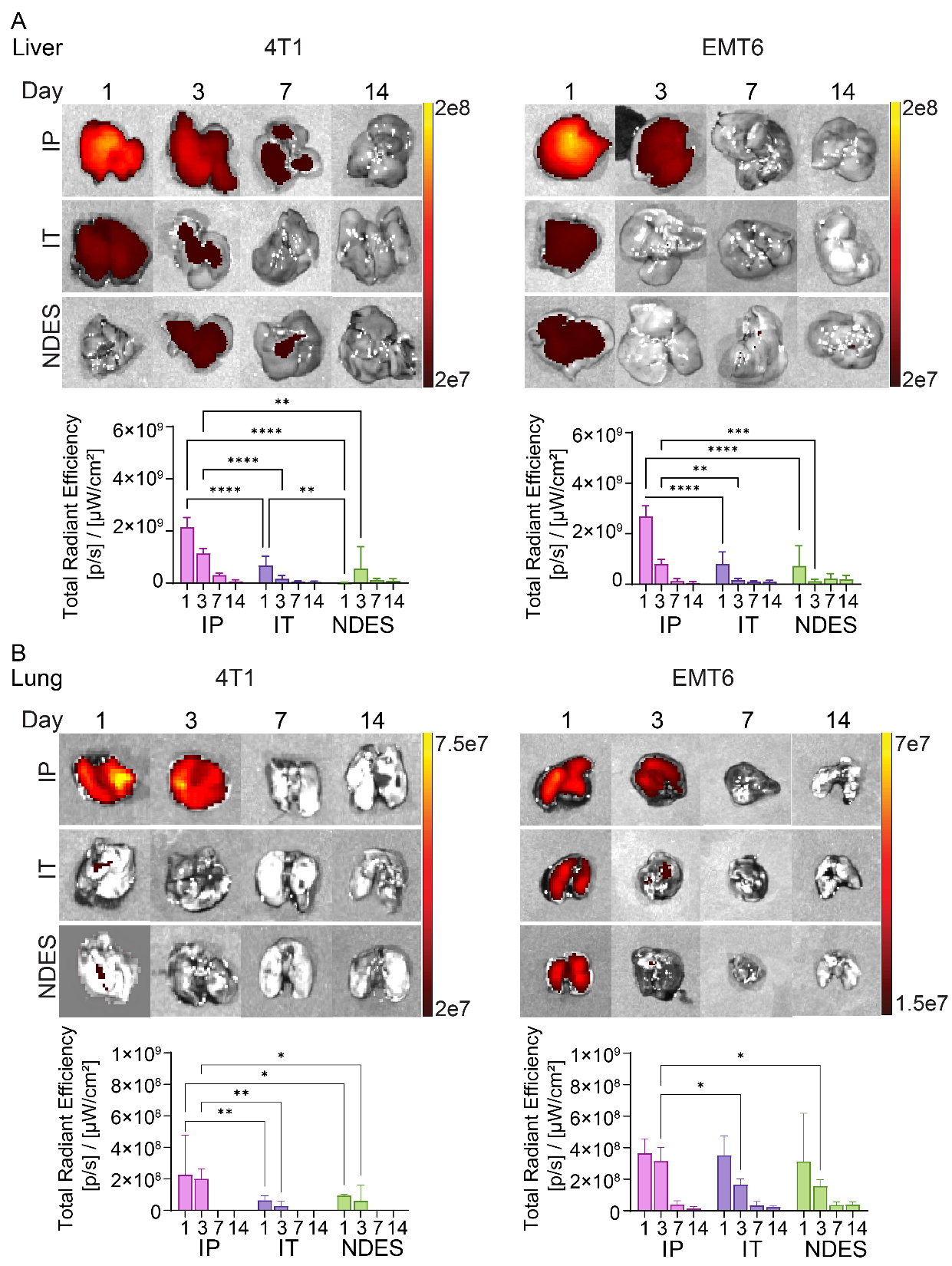


**sFig 6.** Ex vivo fluorescence imaging analysis of the organs from 4T1 (left) and EMT6 (right) mice over 14 days after αPD-L1-AF700. A) representative livers from each time points and bar graph depicts radiance signal measured. B) representative lungs from each time points and bar graph depicts radiance signal measured 2way ANOVA was performed for statistical analysis. p<0.05, *; p<0.005, **; p<0.001, ***; p<0.0001, ****.


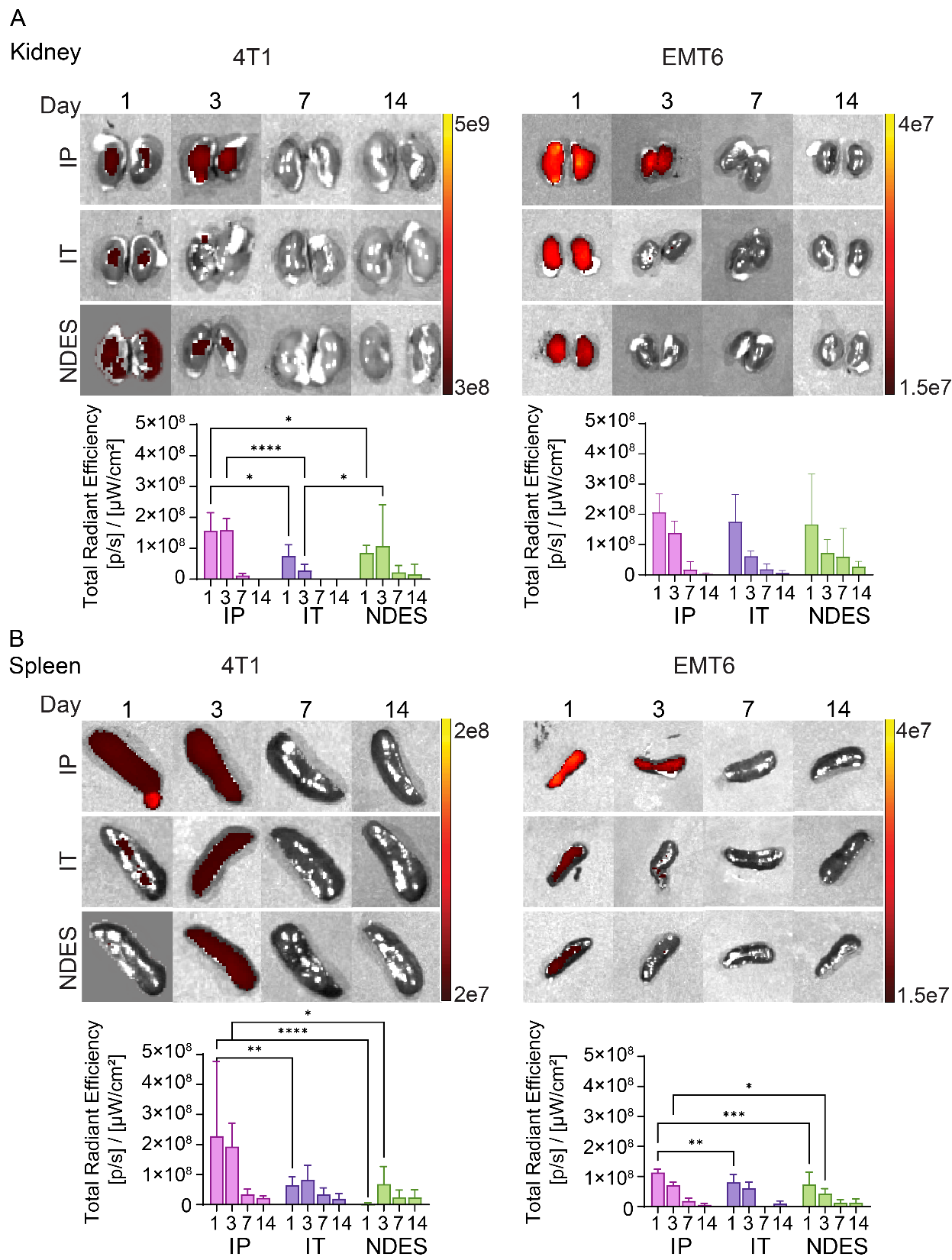


**sFig 7.** Ex vivo fluorescence imaging analysis of the organs from 4T1 (left) and EMT6 (right) mice over 14 days after αPD-L1-AF700. A) representative kidneys from each time points and bar graph depicts radiance signal measured. B) representative spleens from each time points and bar graph depicts radiance signal measured 2way ANOVA was performed for statistical analysis. p<0.05, *; p<0.005, **; p<0.001, ***; p<0.0001, ****.


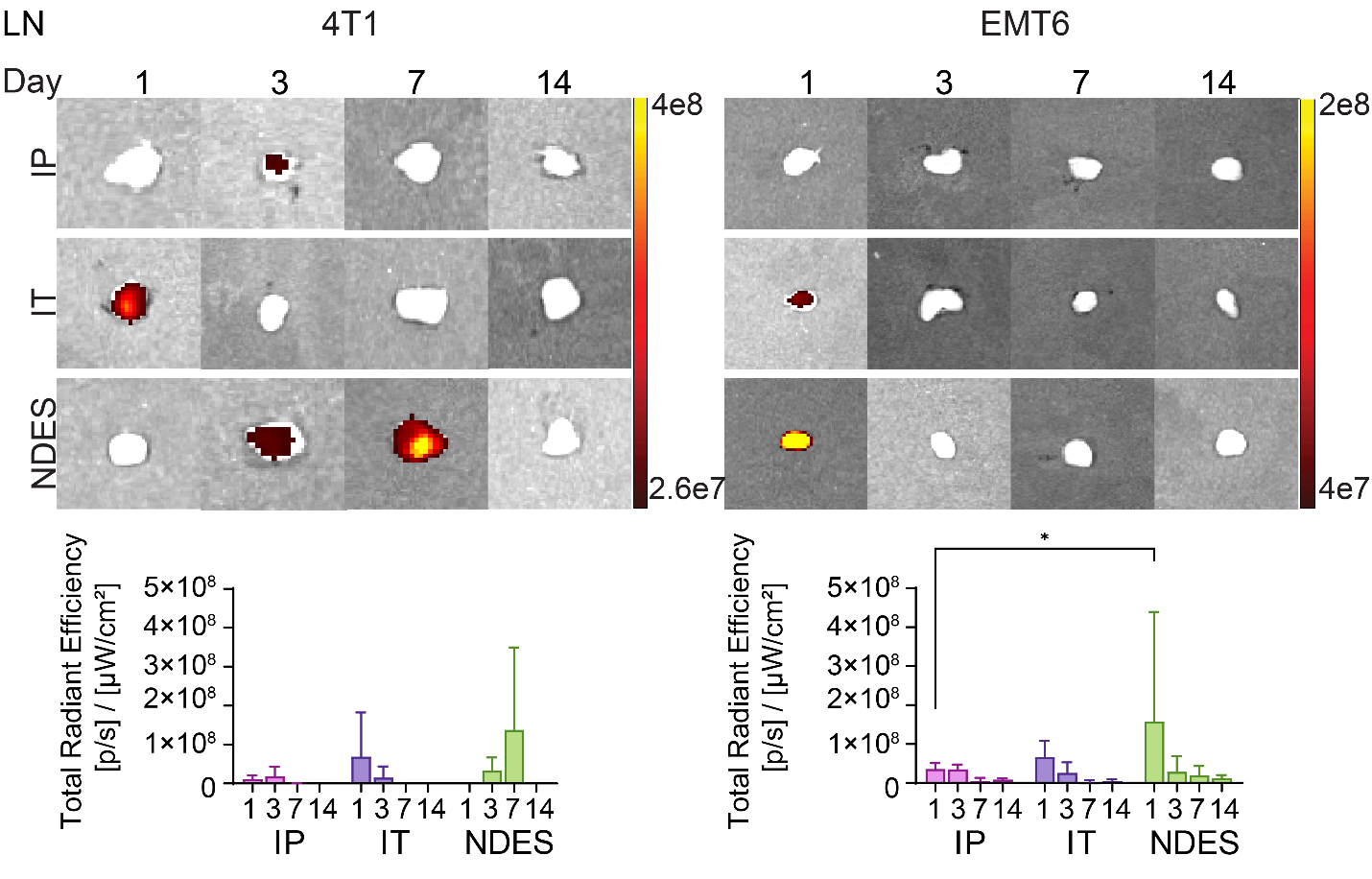


**sFig 8.** Ex vivo fluorescence imaging analysis of the organs from 4T1 (left) and EMT6 (right) mice over 14 days after αPD-L1-AF700. Representative LNs from each time points and bar graph depicts radiance signal measured. 2way ANOVA was performed for statistical analysis. p<0.05, *; p<0.005, **; p<0.001, ***; p<0.0001, ****.


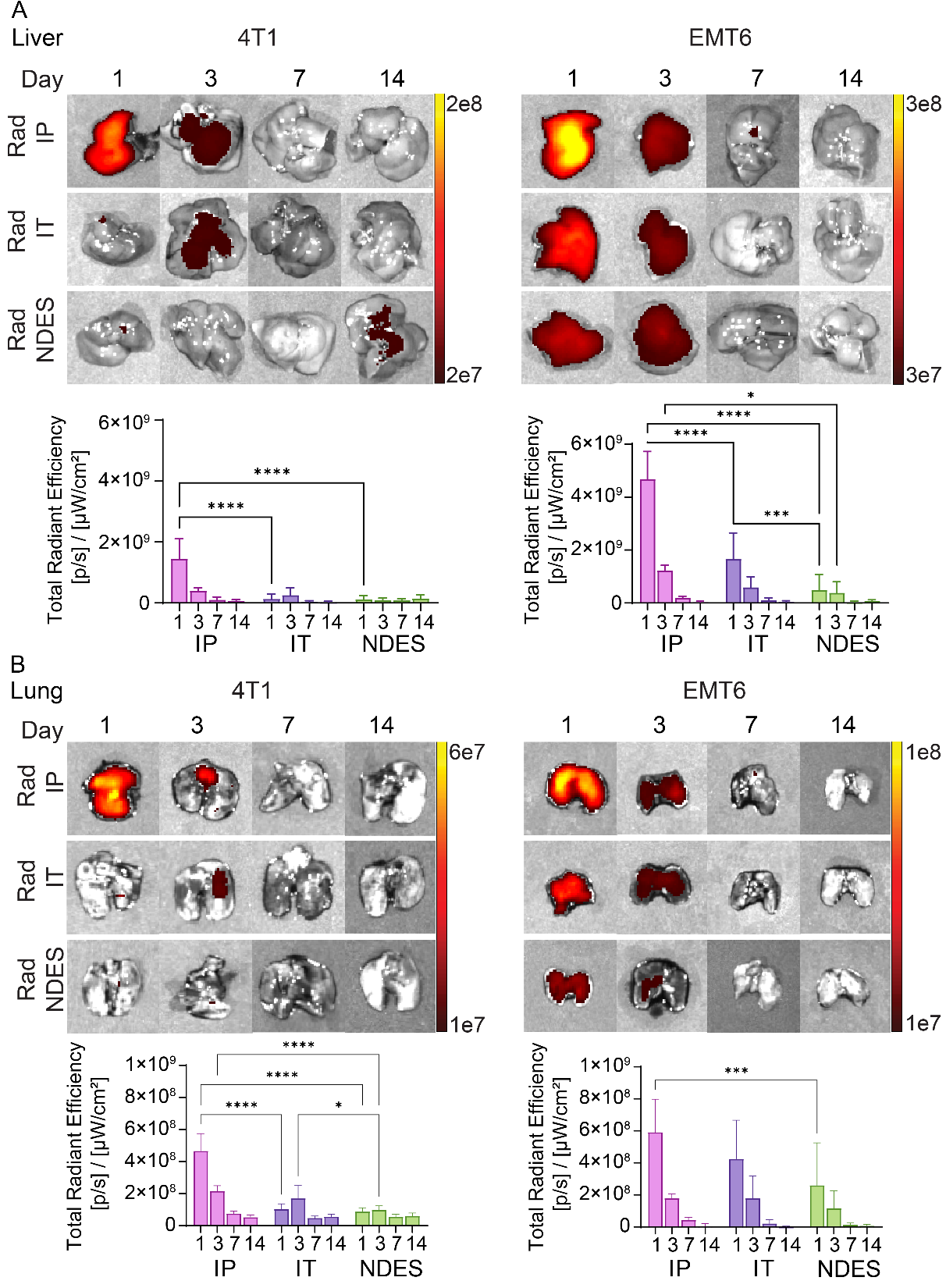


**sFig 9.** Ex vivo fluorescence imaging analysis of the organs from 4T1 (left) and EMT6 (right) mice over 14 days after αPD-L1-AF700. A) representative livers from each time points and bar graph depicts radiance signal measured. B) representative lungs from each time points and bar graph depicts radiance signal measured 2way ANOVA was performed for statistical analysis. p<0.05, *; p<0.005, **; p<0.001, ***; p<0.0001, ****.


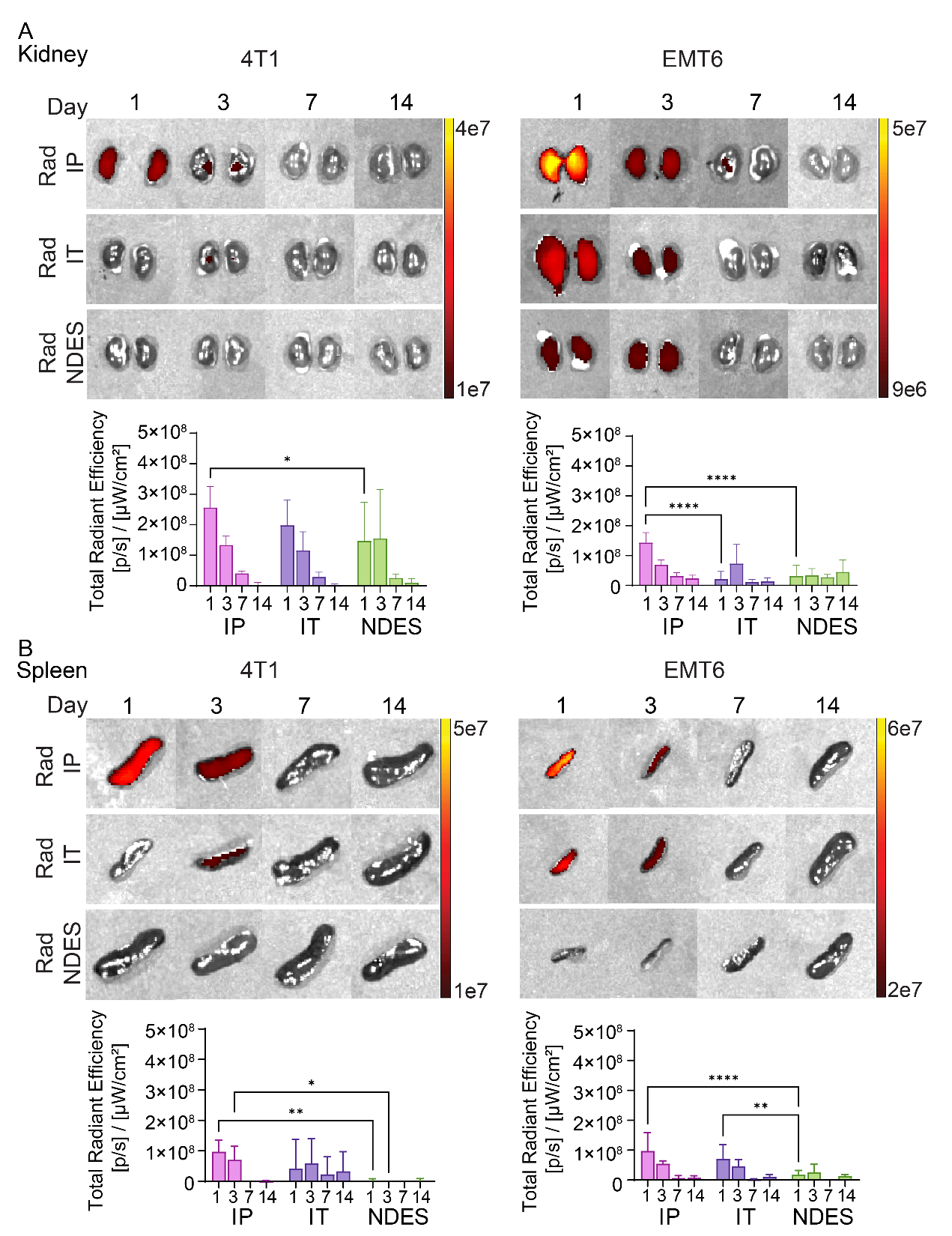


**sFig 10.** Ex vivo fluorescence imaging analysis of the organs from 4T1 (left) and EMT6 (right) mice over 14 days after αPD-L1-AF700. A) representative kidneys from each time points and bar graph depicts radiance signal measured. B) representative spleens from each time points and bar graph depicts radiance signal measured 2way ANOVA was performed for statistical analysis. p<0.05, *; p<0.005, **; p<0.001, ***; p<0.0001, ****.


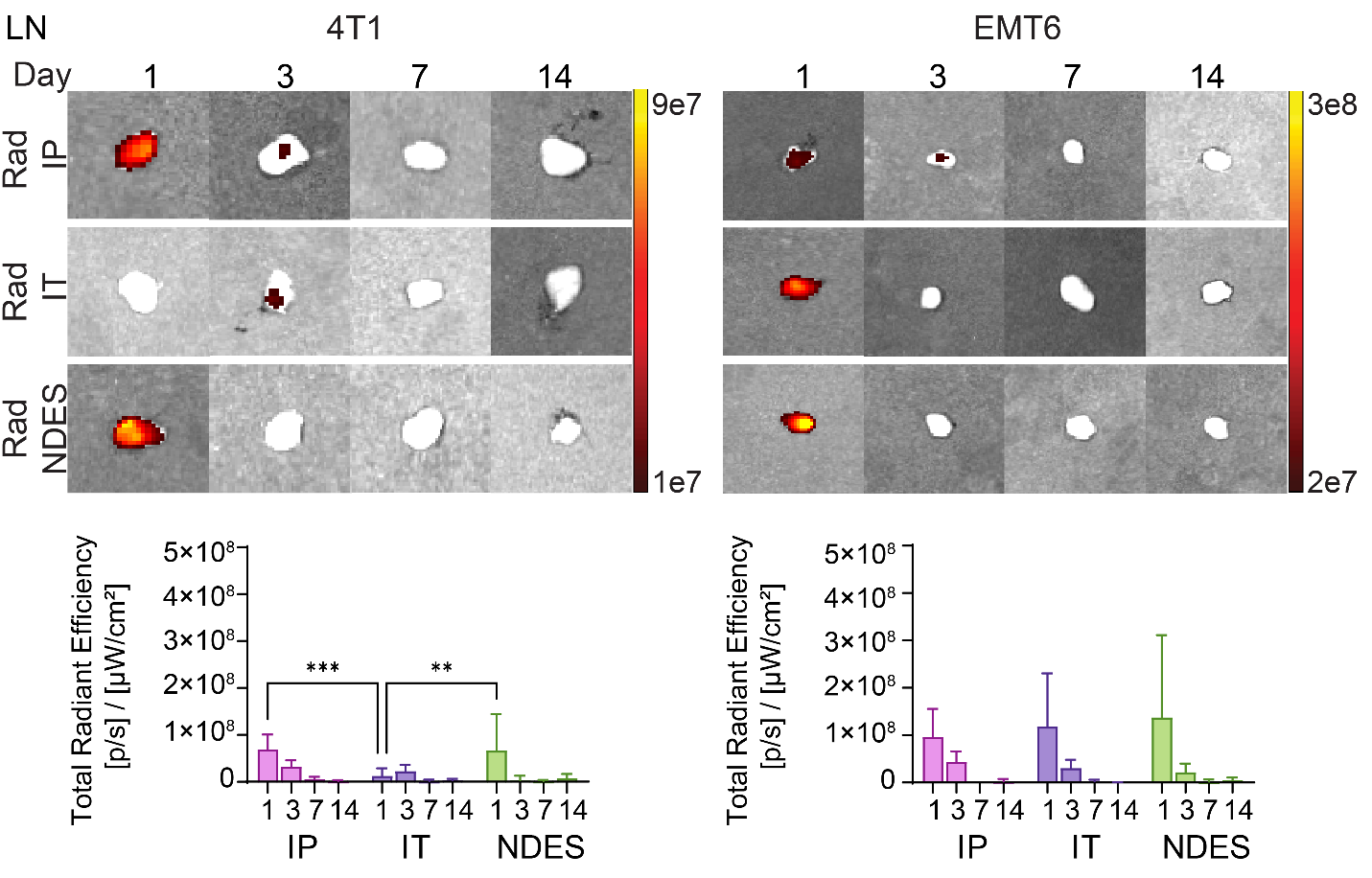


**sFig 11.** Ex vivo fluorescence imaging analysis of the organs from 4T1 (left) and EMT6 (right) mice over 14 days after αPD-L1-AF700. A) representative LNs from each time points and bar graph depicts radiance signal measured. 2way ANOVA was performed for statistical analysis. p<0.05, *; p<0.005, **; p<0.001, ***; p<0.0001, ****.


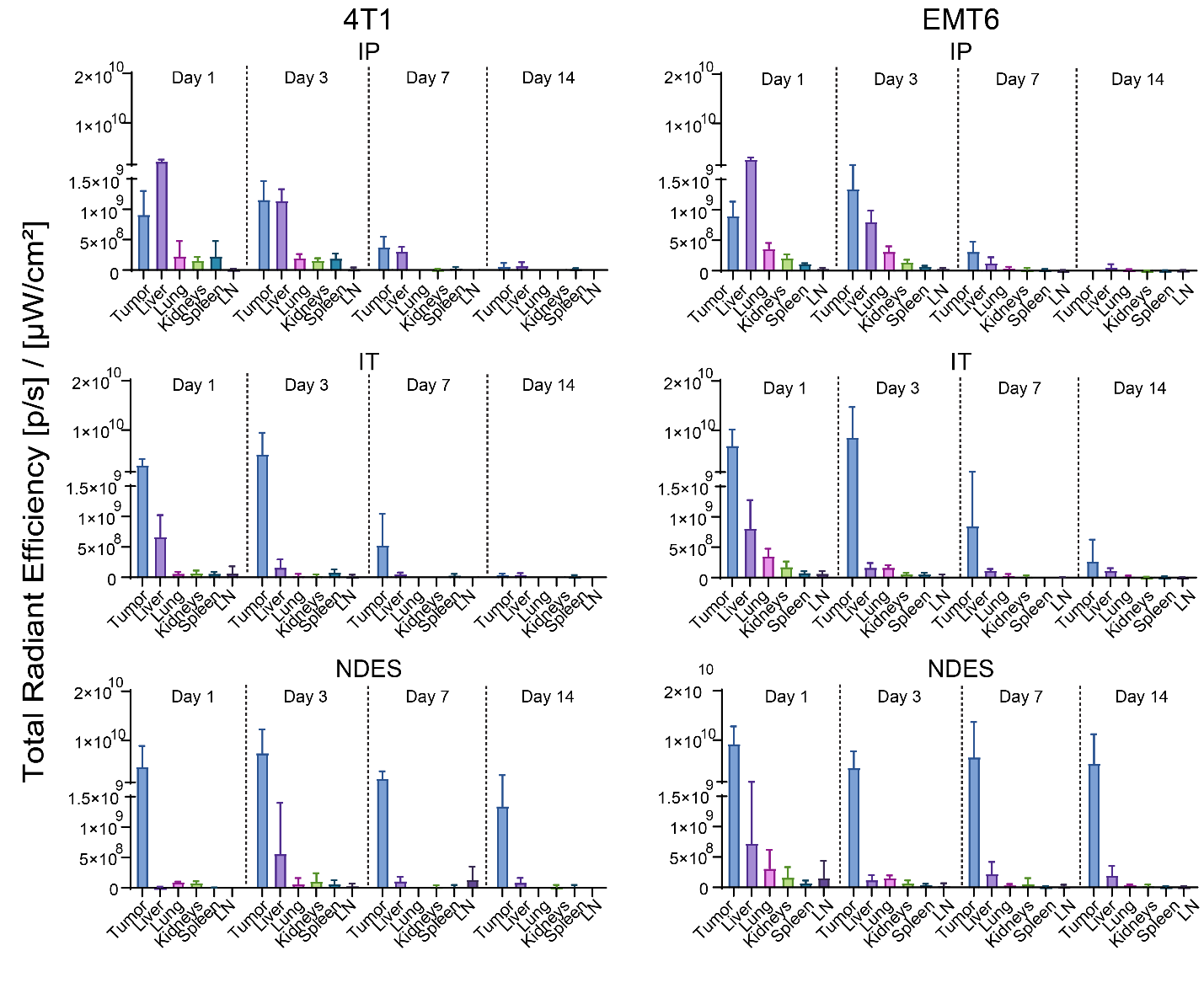


**sFig 12.** Biodistribution of αPDL1-AF700 across different organs over 14 days. Ex vivo fluorescence analysis of the tumor, liver, lung, kidneys, spleen and inguinal LN were analyzed by measuring the radiance signal at the end of sacrificing point of each delivery method. Bar graphs depict radiance signal measured. Each data point represents mean ± STD (n=5-6 per organ).


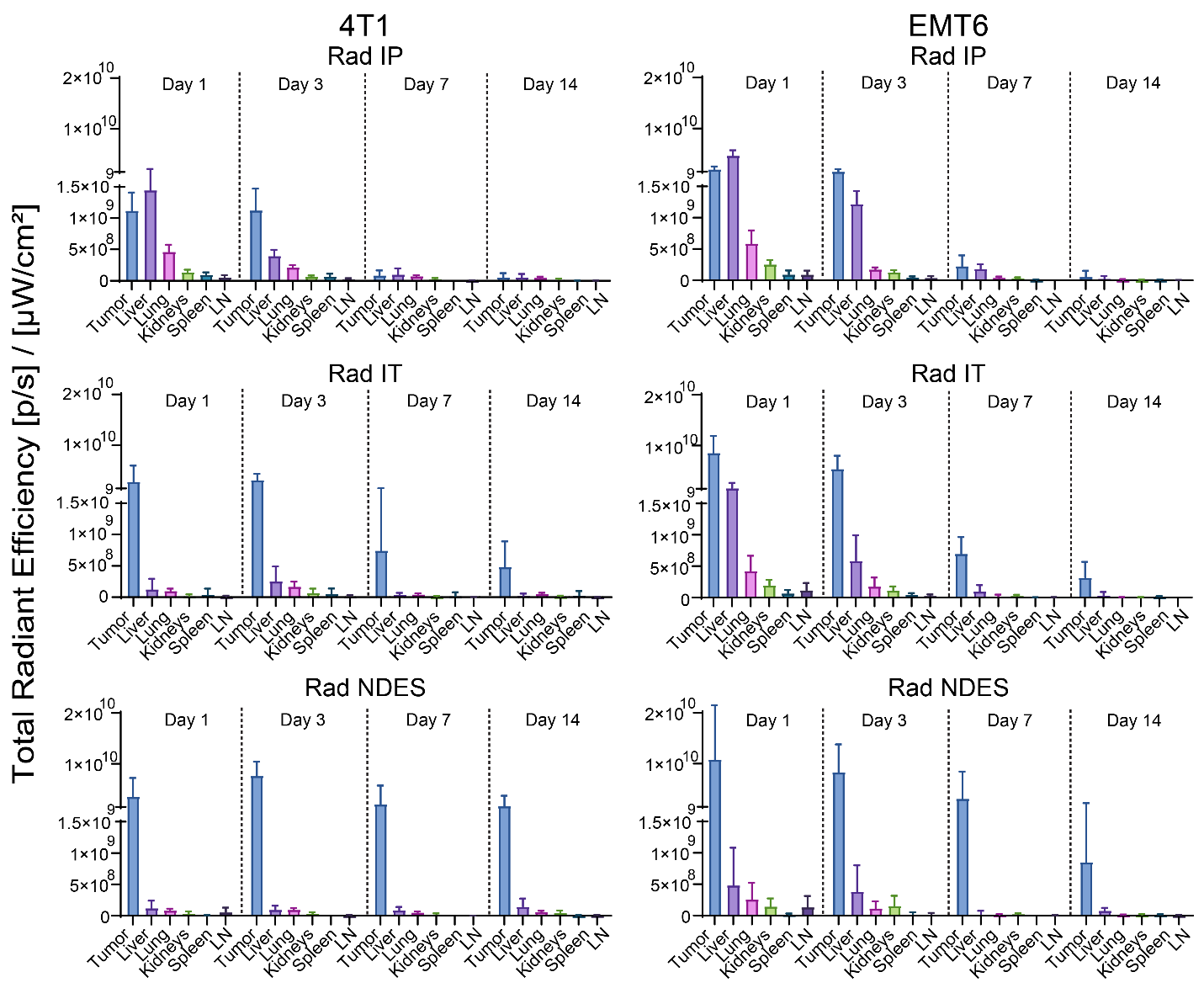


**sFig 13.** Biodistribution of αPDL1-AF700 across different organs over 14 days. Ex vivo fluorescence analysis of the tumor, liver, lung, kidneys, spleen and inguinal LN from radiated mice were analyzed by measuring the radiance signal at the end of sacrificing point of each delivery method. Bar graphs depict radiance signal measured. Each data point represents mean ± STD (n=5-6 per organ).


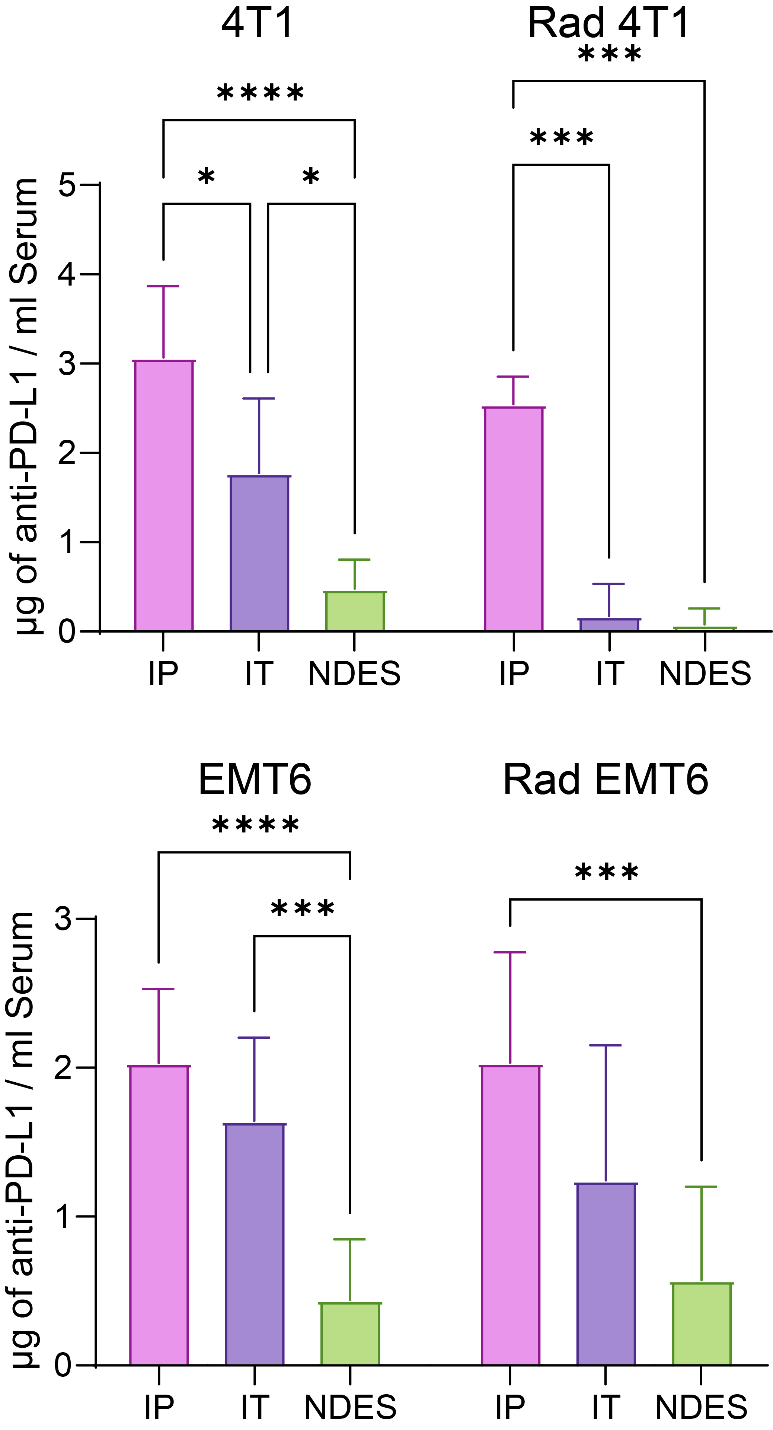


**sFig 14.** ELISA analysis of serum αPD-L1 levels of non-radiated and radiated 4T1 and EMT6 mice 1 day after administered via IP, IT or NDES. Each data point represents mean ± STD (n=6 per group).
